# Practical Preparation of (3*S*)-Hydroxy-5-Phenylpentanoic Acid: Asymmetric Synthesis of (*S*)-Daphneolone and (*S*)-Dihydroyashabushiketol, and Formal Synthesis of (3*S*,5*S*)-Yashabushidiol B

**DOI:** 10.3390/ijms26041476

**Published:** 2025-02-10

**Authors:** So-Yeon Nam, Joungmo Cho, Simon MoonGeun Jung, Hyun-Jun Lee, Hyung Won Ryu, Sei-Ryang Oh, Kee-In Lee

**Affiliations:** 1 Research and Development Center, Molecules & Materials Co., Ltd., Daejeon 34013, Republic of Korea; namsso@hanmail.net; 2 Green Chemistry Division, Korea Research Institute of Chemical Technology, Daejeon 34114, Republic of Korea; jmcho@krict.re.kr; 3 School of Food Biotechnology and Chemical Engineering, Hankyong National University, Anseong 17579, Republic of Korea; 4 Natural Product Research Center, Korea Research Institute of Bioscience and Biotechnology, Cheongju 28116, Republic of Korea; hjlee7@kribb.re.kr (H.-J.L.); ryuhw@kribb.re.kr (H.W.R.); seiryang@kribb.re.kr (S.-R.O.)

**Keywords:** diarylpentanoids, diarylheptanoids, (3*S*)-hydroxy-5-phenylpentanoic acid, Weinreb amide, (*S*)-daphneolone, (*S*)-dihydroyashabushiketol, (3*S*,5*S*)-yashabushidiol B

## Abstract

Many linear diarylpentanoids and diarylheptanoids contain a β-hydroxy ketone or 1,3-diol functionality as the structural motif. Reported herein is the asymmetric synthesis of (*S*)-daphneolone, (*S*)-dihydroyashabushiketol, and formal synthesis of (3*S*,5*S*)-yashabushidiol B as represented examples, employing readily accessible (3*S*)-hydroxy-5-phenylpentanoic acid. The (3*S*)-hydroxy-5-phenylpentanoic acid was conveniently prepared by the aldol addition of (*R*)-acetyloxazolidinone with 3-phenylpropanal affording two diastereomers which were cleanly separated by silica gel column chromatography, followed by the removal of Evans auxiliary of (3′*R*,4*S*)-imide. Then, the (*S*)-acid was converted to Weinreb amide as a privileged acylating agent. Three natural products with the uppermost optical purity were prepared by the treatment of organolithium or organomagnesium reagents, respectively, to the Weinreb amide used in common. We believe that this strategy provides a rapid and convergent method for constructing these classes of molecules of interest.

## 1. Introduction

**Figure 1:**
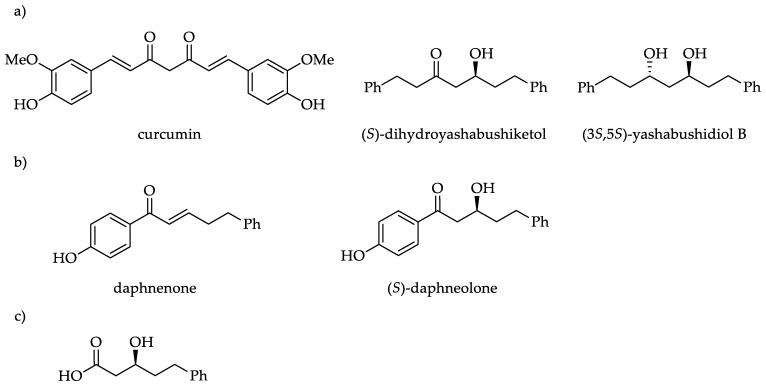
Representative examples of (**a**) diarylheptanoids, (**b**) diarylpentanoids, and (**c**) (3*S*)-hydroxy-5-phenylpentanoic acid as a key building block.

Diarylheptanoids constitute a family of natural plant metabolites with a similar structural pattern, distinctively two aromatic rings tethered by linear seven-carbon bridges. The diarylheptanoids are well known as curcumin analogs and are categorized into linear, cyclic, and dimeric forms [1]. So far, many natural products and their structural modifications account for various medicinal values, such as anti-tumor, anti-inflammatory, antioxidant, and cytoprotective activities [2,3,4]. Among diarylheptanoids, nonracemic yashabushiketols, yashabushidiols, and yashabushitriols have been highly recognized for their absolute stereochemistry and asymmetric synthesis [5], and their structures are depicted in Figure 1a.

Diarylpentanoids also feature an emerging class of bioactive natural products and their structural diversity and array are quite similar to those of diarylheptanoids [6]. As such, these privileged scaffolds serve as platforms for exhibiting anti-proliferative, apoptosis induction, and anti-migration properties [7]. Interestingly, a recent study has revealed a biosynthetic pathway of 2-(2-phenylethyl)chromones catalyzed by a diarylheptanoid-producing polyketide synthase in agarwood, in which diarylpentanoids are the common substrates [8]. Daphneolone is the most widely found in diarylheptanoid-producing species (Figure 1b). Even though racemic synthesis has been reported [9], the availability of natural (*S*)-(+)-daphneolone is rather limited from natural sources [10,11]. To date, no report has been made available in the literature for the asymmetric synthesis of (*S*)-daphneolone. Indeed, the asymmetric synthesis of optically pure β-hydroxy ketones without substituents at the α-position to the carbonyl is still hampered with not only acetate aldol with *N*-acetyloxazolidinones affording nearly 1:1 ratios of aldol adducts [12], but also several asymmetric aldol additions of acetophenone with 3-phenylpropanal providing only moderate enantioselectivity [13,14,15]. The other methods include Cu-catalyzed asymmetric borylation of α,β-unsaturated ketones [16] and the kinetic resolution of β-hydroxy carbonyl compounds through enantioselective dehydration using a cation-binding catalyst [17].

We thus envisioned that significant β-hydroxy ketones would be accessible from enantiopure (3*S*)-hydroxy-5-phenylpentanoic acid as outlined in Figure 1. Here, we would like to report the chiral separation of (3*S*)-hydroxy-5-phenylpentanoic acid through an Evans-type imide by chromatographic separation and then the asymmetric synthesis of (*S*)-daphneolone and other β-hydroxy ketone congeners.

## 2. Results and Discussion

Conventionally, nonracemic 3-hydroxy-5-phenylpentanoic acid has been obtained through synthetic elaboration employing the organocatalytic epoxidation of (*E*)-5-phenyl-2-pentenal and sequential NHC-catalyzed ring-opening and acyl substitution in 94% *ee* [18]. In addition, transition metal-catalyzed aldol addition of *O*-silyl enolate to aldehyde [19], oxycarbonylation of terminal alkene [20], and asymmetric hydroboration of β,γ-unsaturated amide [21] showed more and less than 90% in enantioselectivity. However, the optical resolution of the *rac*-acid with chiral amines still shows poor results [22].

**Scheme 1:**
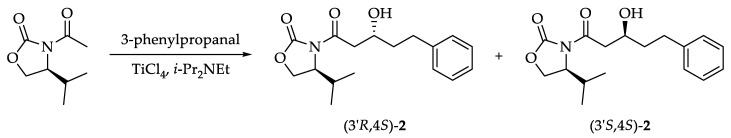
Aldol addition of (4*S*)-oxazolidinone with 3-phenylpropanal.

Although the Evans aldol additions using *N*-acetylated oxazolidinones give generally poor diastereoselectivity, it was suggested that the diastereomeric separation of the resulting Evans imides could offer a facile route for the practical preparation of optically pure β-hydroxy acid. Further transformation to Weinreb amide aids as an excellent acylating agent for organolithium or organomagnesium reagents to generate β-hydroxy ketones [23]. Initially, the aldol addition of (*S*)-3-acetyl-4-isopropyl-2-oxazolidinone and 3-phenylpropanal by TiCl_4_ and *i*-Pr_2_NEt gave two diastereomers, as shown in Scheme 1. HPLC analysis shows (3′*R*,4*S*)-*2*/(3′*S*,4*S*)-**2** = 5.6/1; however, the desired (3′*S*,4*S*)-**2** was a minor amount (vide infra). We thus changed to start from (*R*)-4-isopropyl-2-oxazolidinone.

**Scheme 2:**
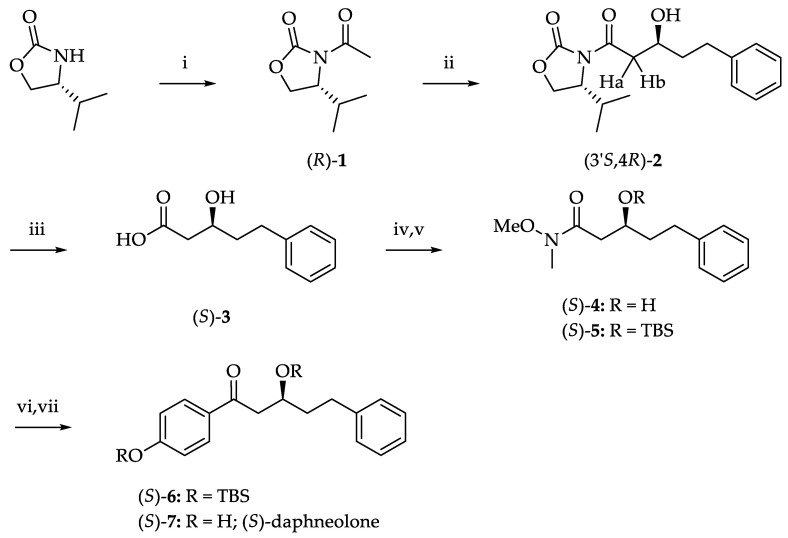
Asymmetric synthesis of (*S*)-daphneolone. Reagent and conditions: (i) AcCl, NaH (60% in mineral oil), THF, 3 h, 93%; (ii) 3-phenylpropanal, TiCl_4_ (1.0 M in CH_2_Cl_2_), *i*-Pr_2_NEt, −78 °C to rt, 12 h, (3′*S*,4*R*)-**2** (49%) and (3′*R*,4*R*)-**2** (9%); (iii) LiOH, H_2_O_2_ (30 wt% in H_2_O), THF/H_2_O, 6 h, 89%; (iv) EDC-HCl, HOBt, MeONHMe-HCl, DMF, 12 h, (*S*)-**4** (83%); (v) TBSCl, imidazole, DMF, 12 h, (*S*)-**5** (90%); (vi) 4-TBSO(C_6_H_4_)Li, −78 °C, 2 h, THF, (*S*)-**6** (43%); (vii) TBAF (1.0 M in THF), THF, 12 h, (*S*)-**7** (82%, 99.9% *ee*).

We then prepared (*R*)-acetyloxazolidinone from the reaction of (*R*)-4-isopropyl-2-oxazolidinone and acetyl chloride with sodium hydride according to the previous procedures [24]. As above, the TiCl_4_-promoted Evans-aldol of (R)-**1** with 3-phenylpropanal afforded two diastereomers which were cleanly separated by silica gel column chromatography as judged by HPLC. Their stereochemistry was assigned by the methylene protons adjacent to the imide carbonyl as observed previously [25,26]. The coupling pattern of the diastereotopic protons serves as a fingerprint: the minor diastereomer (3′*R*,4*R*)-**2** showed an apparent two sets of doublets of doublets (Ha = 3.20 and Hb = 2.99 ppm; *J*_Ha_ = 17.5, 2.6 and *J*_Hb_ = 17.5, 9.2 Hz), whereas the major (3′*S*,4*R*)-**2** appeared distinctively as a doublet for the signals (Hab = 3.11 ppm; *J*_Hab_ = 6.1 Hz). The isolated ratio of (3′*S*,4*R*)-**2**/(3′*R*,4*R*)-**2** was 5.4/1, interestingly, the diastereoselectivity was much higher than expected as observed in the other aliphatic aldehydes [12,25]. Evans auxiliary was removed satisfactorily using LiOH/H_2_O_2_ to furnish (3*S*)-hydroxy-5-phenylpentanoic acid, (*S*)-**3** in 89% yield [27]. The *ee* value of (*S*)-**3** was determined as its methyl ester and showed 98.5% *ee*. Weinreb amide (*S*)-**4** was conveniently prepared from the corresponding acid via EDC coupling with *N*,*O*-dimethylhydroxylamine and then transformed to TBS ether (S)-**5** at the standard conditions, as illustrated in Scheme 2.

For the final installation of β-hydroxy ketones, we retained just the selection of the corresponding organolithium or organomagnesium reagent to react with Weinreb amide used in common. The addition of three equivalents of 4-TBSO(C_6_H_4_)Li (prepared from 4-TBSO(C_6_H_4_)Br and two equivalents of *t*-BuLi) to the amide (*S*)-**5** gave the ketone (*S*)-**6**, where the transmetalation of the bromide was poor with *n*-BuLi. When *n*-BuLi was used, the transmetalation was sluggish and the reaction was not completed. The silyl group of (*S*)-**6** was removed by treatment with TBAF to furnish optically pure (*S*)-daphneolone (*S*)-**7**, 99.9% *ee* judged by HPLC.

**Scheme 3:**
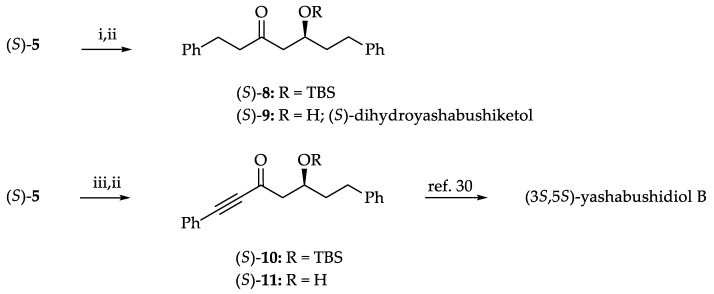
Asymmetric synthesis of (*S*)-dihydroyashabushiketol and formal synthesis of (3*S*,5*S*)-yashabushidiol B. Reagent and conditions: (i) PhCH_2_CH_2_MgCl (1.0 M in THF), −78 °C to rt, 12 h, (*S*)-**8** (93%); (ii) 1N HCl, EtOH, 12 h, (*S*)-**9** (86%, 99.7% *ee*); (*S*)-**11** (83%, 99.9% *ee*); (iii) PhC≡CMgBr (1.0 M in THF), −78 °C to rt, 12 h, (*S*)-**10** (79%).

Previously, (*S*)-dihydroyashabushiketol was obtained through the pre-construction of enantioenriched dihydroisoxazoles [28,29], and (+)-, (−)-yashabushidiol B mainly employing the 1,3-asymmetric induction of chiral β-hydroxy carbonyl compounds [30,31] or -epoxides [32]. In this approach, we only engaged the addition of organometallics to the amide and chose commercially available Grignard reagents. The Grignard reactions of phenethylmagnesium chloride and phenylethynylmagnesium bromide to (*S*)-**5** smoothly proceeded to yield (*S*)-**8** in 93% and (*S*)-**10** in 79% yield, respectively (Scheme 3). However, when the alcohol (*S*)-**4** was used the Grignard reactions were feeble, and this is quite contrasting to that of the lithium analog for (*S*)-**10** [30].

At the final stage, we encountered a problem with the deprotection of the TBS ethers with TBAF in THF. The reactions were very complex likely due to retro-aldol and dehydration, shortly after desilylation. Furthermore, an intramolecular alkyne cyclization of (*S*)-**10** mediated by TBAF may be possible [33]. The problem was solved by the use of 1N HCl in EtOH to furnish optically pure (*S*)-dihydroyashabushiketol (*S*)-**9** (99.7% *ee*) and (*S*)-**11** (99.9% *ee*), respectively, in which the latter was used for the synthesis of (3*S*,5*S*)-yashabushidiol B [30].

## 3. Materials and Methods

### 3.1. General

Unless otherwise specified, all reactions were carried out under a nitrogen atmosphere in oven-dried glassware with magnetic stirring. All reagents and anhydrous solvents were purchased and used without any further purification. Thin layer chromatography (TLC) was performed using Merck aluminum foil-backed sheets pre-coated with Kieselgel 60F254. Column chromatography refers to chromatography on Merck Silica gel C60 (40–60 µm). The yield refers to isolated yield.

Melting points were determined in a capillary and were uncorrected. NMR spectra were recorded on a Bruker DPX-400, a Bruker AVANCE AV400, a Bruker DPX-500, or a Bruker AMX-500 spectrometer. The experiments were performed at 400 MHz for ^1^H and 100 MHz for ^13^C, except where otherwise specified. Chemical shifts (δ) are reported in parts per million (ppm) from tetramethylsilane with the undeuterated solvent resonance as the internal standard. Mass spectra (*m/z*) were recorded on a Waters ACQUITY UPLC H-Class/SQD2 Mass Spectrometer in Electrospray Ionization (ESI) or Atmospheric Pressure Chemical Ionization (APCI), and HRMS were recorded on a JEOL JMS-700 in Chemical Ionization (CI), Electron Impact (EI), or Fast atom bombardment (FAB) modes. Optical rotations were determined on a Perkin-Elmer 241 polarimeter in a 1 dm cell. Concentrations are given in g/100 mL. The determination of values of enantiomeric excess (*ee*) and diastereomeric excess (*de*) was performed by high-performance liquid chromatography (HPLC) using Thermo/Dionex UltiMate 3000 HPLC System equipped with Chiralcel OJ-H column (4.6 × 250 mm), and analytical conditions were specified.

### 3.2. Preliminary Result

#### Aldol Addition of (S)-3-Acetyl-4-Isopropyl-2-Oxazolidinone with 3-Phenylpropanal (See, Scheme 1)

The aldol addition of (*S*)-3-acetyl-4-isopropyl-2-oxazolidinone and 3-phenylpropanal with TiCl_4_ (1.0 M in CH_2_Cl_2_) and *i*-Pr_2_NEt gave two diastereomers and HPLC analysis shows (3′*R*,4*S*)-**2**/(3′*S*,4*S*)-**2** = 5.6/1. These were readily separated by silica gel column chromatography. Their stereochemistry was judged by the diastereotopic methylene protons adjacent to the imide carbonyl as observed previously [25,26]. However, the desired product, (3′*S*,4*S*)-**2** was a minor amount. HPLC: Chiralcel OJ-H column, IPA/hexane = 30/70, 1.0 mL/min, 210 nm; t_1_ = 11.6 [(3′*R*,4*S*)-**2**], t_2_ = 13.3 min [(3′*S*,4*S*)-**2**].

### 3.3. Synthesis of (S)-3-Hydroxy-5-Phenylpentanoic Acid

#### 3.3.1. Preparation of (*R*)-3-Acetyl-4-Isopropyl-2-Oxazolidinone, (*R*)-**1** [24]

According to the previous procedures, (*R*)-**1** was prepared from (*R*)-4-isopropyl-2-oxazolidinone and acetyl chloride with sodium hydride.

(*R*)-**1:** 92.8% yield (6.35 g); ^1^H NMR (400 MHz, CDCl_3_) δ 4.48–4.37 (m, 1H), 4.31–4.17 (m, 2H), 2.54 (s, 3H), 2.45–2.35 (m, 1H), 0.92 (d, *J* = 7.0 Hz, 3H), 0.88 (d, *J* = 6.9 Hz, 3H); ^13^C NMR (100 MHz, CDCl3): δ 170.2, 154.3, 63.3, 58.3, 28.3, 23.8, 17.9, 14.6.

#### 3.3.2. Aldol Addition of (*R*)-**1** with 3-Phenylpropanal

To a stirred solution of (*R*)-**1** (5.0 g, 29.2 mmol) in methylene chloride (150 mL) at −78 °C under nitrogen, 1.0 M TiCl_4_ in methylene chloride (58.4 mL, 58.4 mmol) was added. After 10 min, *i*-Pr_2_NEt (9.93 mL, 58.4 mmol) was added and stirred for 1 h at the same temperature, followed by 3-phenylpropanal (7.68 mL, 58.4 mmol). The reaction mixture was maintained at −78 °C for 5 h and warmed to room temperature overnight, and then a saturated ammonium chloride solution (50 mL) was added. The organic layer was washed with water (50 mL) and then brine (50 mL), dried over anhydrous magnesium sulfate, and concentrated in vacuo to give the diastereomers. They were separated by silica gel chromatography eluting with 15% EtOAc/petroleum ether to give (3′*S*,4*R*)-**2** as the major and (3′*R*,4*R*)-**2**, respectively, in 58.2% overall yield.

(3′*S*,4*R*)-**2**: 48.9% yield (4.36 g); 99.8% *de*; ^1^H NMR (400 MHz, CDCl_3_) δ 7.32–7.26 (m, 2H), 7.24–7.11 (m, 3H), 4.49–4.39 (m, 1H), 4.33–4.18 (m, 2H), 4.07 (dt, *J* = 9.8, 5.9 Hz, 1H), 3.11 (d, *J* = 6.1 Hz, 2H), 3.05 (d, *J* = 4.4 Hz, 1H), 2.89–2.79 (m, 1H), 2.73 (ddd, *J* = 13.8, 9.2, 7.1 Hz, 1H), 2.45–2.31 (m, 1H), 1.97–1.86 (m, 1H), 1.85–1.73 (m, 1H), 0.93 (d. *J* = 7.0 Hz, 3H), 0.88 (d, *J* = 6.9 Hz, 3H); ^13^C NMR (100 MHz, CDCl_3_): δ 172.7, 154.1, 141.7, 128.5, 128.4, 125.8, 67.2, 63.5, 58.4, 42.5, 38.1, 31.7, 28.4, 17.9, 14.7; HRMS (EI): *m/z* [M]+ calcd for C_17_H_23_NO_4_ 305.1627, observed 305.1628; HPLC: Chiralcel OJ-H column, IPA/hexane = 30/70, 1.0 mL/min, 210 nm; t_1_ = 12.7 [(3′*R*,4*R*)-**2**], t_2_ = 13.9 min [(3′*S*,4*R*)-**2**].

(3′*R*,4*R*)-**2**: 9.3% yield (0.83 g); 92.4% *de*; ^1^H NMR (400 MHz, CDCl_3_): δ 7.28 (dd, *J* = 12.6, 5.2 Hz, 2H), 7.20 (dd, *J* = 15.8, 7.3 Hz, 3H), 4.49–4.39 (m, 1H), 4.34–4.19 (m, 2H), 4.16–4.05 (m, 1H), 3.20 (dd, *J* = 17.5, 2.6 Hz, 1H), 2.99 (dd, *J* = 17.5, 9.2 Hz, 1H), 2.95 (d, *J* = 4.7 Hz, 1H), 2.89–2.79 (m, 1H), 2.73 (ddd, *J* = 13.8, 9.2, 7.1 Hz, 1H), 2.42–2.30 (m, 1H), 1.96–1.85 (m, 1H), 1.85–1.73 (m, 1H), 0.92 (d, *J* = 7.0 Hz, 3H), 0.88 (d, *J* = 6.9 Hz, 3H); ^13^C NMR (100 MHz, CDCl_3_): δ 172.7, 154.0, 141.7, 128.5, 128.4, 125.8, 67.1, 63.5, 58.4, 42.6, 38.0, 31.7, 28.4, 17.9, 14.7; HRMS (EI): *m/z* [M]^+^ calcd for C_17_H_23_NO_4_ 305.1627, observed 305.1630; HPLC: Chiralcel OJ-H column, IPA/hexane = 30/70, 1.0 mL/min, 210 nm; t_1_ = 12.6 [(3′*R*,4*R*)-**2**], t_2_ = 13.8 min [(3′*S*,4*R*)-**2**].

#### 3.3.3. Synthesis of (*S*)-3-Hydroxy-5-Phenylpentanoic Acid, (*S*)-**3** [20]

A solution of the imide (3′*S*,4*R*)-**2** (2.50 g, 8.18 mmol) in THF (80 mL) and water (20 mL) was treated with LiOH (310 mg, 12.9 mmol) and 30 wt% H_2_O_2_ in water (3.80 mL, 37.2 mmol) at 0 °C. After stirring at the same temperature for 6 h, the reaction was quenched by adding sat. NaHCO_3_ solution (50 mL) and the organic solvent was removed. The aqueous layer was washed with CH_2_Cl_2_ (3 × 20 mL), acidified with 1N HCl adjusted to *p*H = 2, and extracted with Et_2_O (3 × 30 mL). The combined organic phases were dried over anhydrous Na_2_SO_4_, and concentrated under reduced pressure to give (*S*)-**3** as a white solid.

(*S*)*-***3**. 88.7% (1.41 g); mp = 134–126 °C; [α]_D_^24^ = −43.4 (*c* 1.06, MeOH); ^1^H NMR (400 MHz, DMSO-*d*_6_): δ 7.28 (t, *J* = 7.4 Hz, 2H), 7.23–7.08 (m, 3H), 3.82 (m, 1H), 3.35 (br s, 1H), 2.51 (m, 1H), 2.37 (m, 1H), 2.36 (dd, *J* = 14.8, 5.0 Hz, 1H), 2.27 (dd, *J* = 14.8, 8.0 Hz, 1H), 1.67–1.63 (m, 2H); ^13^C NMR (100 MHz, DMSO-*d*_6_): δ 173.4, 142.6, 128.8, 128.7, 126.0, 67.0, 43.1, 39.2, 31.7; MS (ESI): *m/z* 194.1 [M]^+^.

The optical purity of (*S*)-**3** was determined after derivatization to its corresponding methyl ester (MeI, Cs_2_CO_3_, DMF; 87%) and shows 98.5% *ee*.

(*S*)-**3**-OMe. HPLC: Chiralcel OJ-H column, IPA/hexane = 30/70, 1.0 mL/min, 210 nm; t_1_ = 5.5 [(*S*)-**3**-OMe], t_2_ = 8.2 min [(*R*)-**3**-OMe].

#### 3.3.4. Synthesis of Weinreb Amide, (*S*)-**4** [30]

A solution of (*S*)-**3** (300 mg, 1.54 mmol) in DMF (6 mL) was treated with EDC-HCl (445 mg, 2.32 mmol), HOBt (313 mg, 2.32 mmol), MeONHMe-HCl (313 mg, 2.32 mmol), and Et_3_N (0.64 mL, 4.62 mmol). The reaction was stirred at room temperature overnight. After completion, the reaction mixture was diluted with EtOAc (50 mL), and the combined were successively washed with sat. NaHCO_3_ solution, water, and then brine. The organic layer was dried over anhydrous MgSO_4_ and concentrated in vacuo. The crude was purified by silica gel column chromatography (5% MeOH/CH_2_Cl_2_) to provide (S)-4 as a colorless oil.

(*S*)-**4**. 83.2% (304 mg); [α]_D_^24^ = +33.6 (*c* 0.52, CHCl_3_); 1H NMR (400 MHz, CDCl_3_): δ 7.29–7.15 (m, 5H), 4.03 (m, 1H), 3.86 (d, *J* = 2.6 Hz, 1H), 3.66 (s, 3H), 3.18 (s, 3H), 2.83 (td, *J* = 9.3, 5.0 Hz, 1H), 2.74–2.63 (m, 1H), 2.51 (dd, *J* = 14.6, 5.8 Hz, 1H), 1.87 (td, *J* = 9.6, 4.4 Hz, 1H), 1.76 (m, 1H); ^13^C NMR (100 MHz, CDCl_3_): δ 173.8, 142.0, 128.5, 128.3, 125.8, 67.2, 61.2, 38.2 (2C), 31.8 (2C); HRMS (EI): *m*/*z* [M]+ calcd for C_13_H_19_NO_3_ 237.1365, observed 237.1366.

#### 3.3.5. Preparation of TBS Ether, (*S*)-**5**

TBSCl (1.83 g, 12.1 mmol) and imidazole (1.03 g, 15.2 mmol) were added to a stirred solution of (*S*)-**4** (2.40 g, 10.1 mmol) in DMF (30 mL). The reaction mixture was stirred at room temperature overnight and was quenched with sat. NH_4_Cl solution (150 mL). The resulting combination was extracted with EtOAc (3 × 50 mL) and was washed with water (50 mL) and brine (50 mL), dried over anhydrous MgSO_4_, and concentrated in vacuo. The crude was purified by silica gel column chromatography (EtOAc/Hex = 1/9) to provide (*S*)-**5** a colorless oil.

(*S*)-**5**. 89.5% (3.18 g); ^1^H NMR (400 MHz, CDCl_3_): δ 7.29–7.15 (m, 5H), 4.31 (quintet, *J* = 6.4 Hz, 1H), 3.68 (s, 3H), 3.17 (s, 3H), 2.73–2.64 (m, 3H), 2.46 (dd, *J* = 14.8, 5.6 Hz, 1H), 1.87–1.77 (m, 2H), 0.89 (s, 9H), 0.08 (s, 3H), 0.04 (s, 3H); ^13^C NMR (100 MHz, CDCl_3_): δ 172.3, 142.4, 128.3, 127.7, 69.1, 61.3, 39.7, 39.6, 31.9, 31.5, 25.9, 18.0, −4.58, −4.69; MS (ESI): *m*/*z* 374.2 [M + Na]^+^.

### 3.4. Synthesis of (S)-Daphneolone

#### 3.4.1. Preparation of Rac-Daphneolone [9]

According to literature procedures, *rac*-daphneolone was prepared from 4-TMSO-PhCOMe with 3-phenylpropanal through aldol addition (LDA, −78 °C, THF).

*rac*-**7**. HPLC: Chiralcel OJ-H column, IPA/hexane = 25/75, 1.0 mL/min, 254 nm; t_1_ = 10.2 (*S*), t_2_ = 12.5 min (*R*).

#### 3.4.2. Isolation of (*S*)-(+)-Daphneolone [10,11]

Natural daphneolone was isolated from *Daphne giraldii* callus according to the literature procedures and identified as (*S*)-(+)-daphneolone.

#### 3.4.3. Asymmetric Synthesis of (*S*)-Daphneolone

##### Synthesis of β-Hydroxyketone, (*S*)-**6**

To a stirred solution of 4-Br-PhOTBS (1.94 g, 6.75 mmol) in THF (8 mL) at −78 °C under nitrogen, 1.7M *t*-BuLi in hexane (7.7 mL, 13.1 mmol) was added dropwise. The solution was stirred at the same temperature for 30 min, then placed at room temperature, and stirred for 30 min. (*S*)-**5** (766 mg, 2.18 mmol) in THF (7 mL) was added dropwise to this solution cooled again to −78 °C. The resulting combination was kept at −78 °C and stirred for over 2 h, then poured into sat. NH_4_Cl solution (25 mL). The mixture was diluted with 20% EtOAc/hexane (100 mL) and the layers were separated. The aqueous layer was extracted with 20% EtOAc/hexane (3 × 30 mL), the combined organic layers were washed successively with water (30 mL) and brine (30 mL), dried over anhydrous MgSO_4,_ and concentrated in vacuo. The crude was purified by silica gel column chromatography (2% EtOAc/hexane) to provide (*S*)-**6** as a colorless oil.

(*S*)-**6**. 42.9% (467 mg); ^1^H NMR (400 MHz, CDCl_3_): δ 7.94 (d, *J* = 8.7 Hz, 2H), 7.36–7.24 (m, 5H), 6.93 (d, *J* = 8.6 Hz, 2H), 4.49 (quintet, *J* = 6.2 Hz, 1H), 3.28 (dd, *J* = 15.2, 6.8 Hz, 1H), 2.99 (dd, *J* = 15.2, 5.6 Hz, 1H), 2.80–2.77 (m, 2H), 1.97–1.90 (m, 2H), 1.06 (s, 9H), 0.91 (s, 9H), 0.30 (s, 6H), 0.13 (s, 3H), 0.07 (s, 3H); ^13^C NMR (100 MHz, CDCl_3_): δ 197.8, 160.2, 142.3, 131.3, 130.6, 128.4, 128.3, 125.7, 119.8, 69.4, 45.6, 31.4, 25.8, 25.6, 18.2, 18.0, −4.36, −4.60, −4.67; MS (ESI): *m/z* 521.3 [M + Na]^+^.

##### Synthesis of (*S*)-Daphneolone, (*S*)-**7**

To a cold (0 °C) solution of (*S*)-**6** (270 mg, 0.54 mmol) in anhydrous THF (10 mL) was added 1M TBAF in THF (2.16 mL, 2.16 mmol), and the mixture was stirred overnight allowing the mixture to warm to room temperature. The reaction was quenched by the dropwise addition of sat. NH_4_Cl solution (5 mL) and the organic solvent was removed. The aqueous layer was extracted with EtOAc (3 × 10 mL). The combined organic phases were dried over anhydrous Na_2_SO_4_, and concentrated under reduced pressure to give a residue. The crude product was purified by flash column chromatography (5–20% EtOAc/hexane) to give (*S*)-**7** as a white solid.

(*S*)-**7**. 82.2% (120 mg); mp 118–120 °C; [α]_D_^24^ = +5.7 (*c* 0.16, MeOH); 99.9% *ee*; ^1^H NMR (400 MHz, CD_3_OD): δ 7.86 (d, *J* = 8.8 Hz, 2H), 7.24–7.13 (m, 5H), 6.82 (d, *J* = 8.8 Hz, 2H), 4.16 (septet, *J* = 4.6 Hz, 1H), 3.12 (dd, *J* = 15.8, 7.9 Hz, 1H), 3.00 (dd, *J* = 15.8, 4.5 Hz, 1H), 2.76 (m, 1H), 2.62 (m, 1H), 1.84–1.80 (m, 2H); ^13^C NMR (100 MHz, CD_3_OD): δ 198.6, 162.5, 142.0, 130.6, 129.0, 128.0, 127.9, 125.3, 114.8, 67.5, 45.1, 38.8, 31.5; HRMS (EI): *m/z* [M]^+^ calcd for C_17_H_18_O_3_ 270.1256, observed 270.1250.

### 3.5. Synthesis of (S)-Dihydroyashachecked, OKbushiketol and Formal Synthesis of (3S,5S)-Yashabushidiol

#### 3.5.1. Typical Procedure for the Synthesis of (*S*)-**10**

To a stirred solution of (*S*)-**5** (300 mg, 0.85 mmol) in THF (3 mL) at −78 °C under nitrogen was added dropwise 2.56 mL of phenylethynylmagnesium bromide (1.0 M in THF). The solution was stirred at −78 °C for 30 min, and then placed at room temperature overnight. The solution was poured into sat. NH_4_Cl solution (25 mL) and the mixture was diluted with 20% EtOAc/hexane (30 mL) and the layers were separated. The aqueous layer was extracted with 20% EtOAc/hexane (3 × 10 mL), the combined organic layers were washed successively with water (30 mL) and brine (30 mL), dried over anhydrous MgSO_4,_ and concentrated in vacuo. The crude was purified by silica gel column chromatography (2–5% EtOAc/hexane) to provide (*S*)-**10** as a colorless oil.

(*S*)-**10**: 79.4% yield (265 mg); ^1^H NMR (400 MHz, CDCl_3_) δ 7.55 (dd, *J* = 8.3, 1.3 Hz, 2H), 7.45 (d, *J* = 7.5 Hz, 1H), 7.39 (dd, *J* = 8.0, 6.7 Hz, 2H), 7.30–7.25 (m, 3H), 7.19 (d, *J* = 7.5 Hz, 3H), 4.41 (dd, *J* = 6.8, 5.6 Hz, 1H), 2.92 (dd, *J* = 14.9, 7.0 Hz, 1H), 2.82 (dd, *J* = 14.9, 5.6 Hz, 1H), 2.75–2.65 (m, 2H), 1.89 (ddt, *J* = 11.3, 9.0, 5.6 Hz, 2H), 0.93–0.85 (m, 9H), 0.12–0.03 (m, 6H); ^13^C NMR (100 MHz, CDCl_3_) δ 186.0, 141.9, 133.0, 130.7, 128.6, 128.4, 128.3, 125.8, 119.9, 91.1, 88.4, 68.6, 53.1, 39.4, 31.3, 25.9, 18.1, −4.4, −4.6; MS (ESI): *m/z* 415.2 (M + Na)^+^.

Similarly, (*S*)-**8** was prepared using phenethylmagnesium chloride (1.0 M in THF).

(*S*)-**8**: 93.1% yield (314 mg); ^1^H NMR (400 MHz, CDCl_3_) δ 7.27 (dd. *J* = 7.4, 6.0 Hz, 5H), 7.17 (dt, *J* = 12.8, 4.4 Hz, 6H), 4.24 (dd, *J* = 6.7, 5.6 Hz, 1H), 2.92–2.84 (m, 2H), 2.74 (dd, *J* = 11.2, 4.6 Hz, 2H), 2.64 (ddd, *J* = 12.9, 9.8, 5.2 Hz, 3H), 2.49 (dd, *J* = 15.3, 5.3 Hz, 1H), 1.76 (ddd, *J* = 8.3, 7.2, 4.3 Hz, 2H), 0.88 (s, 9H), 0.07 (s, 3H), 0.01 (s, 3H); ^13^C NMR (100 MHz, CDCl_3_) δ 208.7, 142.1, 141.0, 128.5, 128.4, 128.3, 126.0, 125.8, 68.7, 50.1, 46.1, 39.4, 31.4, 29.5, 25.9, 18.0, −4.5, −4.7; MS (ESI): *m/z* 419.3 (M + Na)^+^.

#### 3.5.2. Typical Procedure for the Synthesis of (*S*)-Dihydroyashabushiketol, (*S*)-**9** [28]

To a solution of (*S*)-**8** (127 mg, 0.32 mmol) in EtOH (10 mL), 1N HCl in water (7 mL) was added and the mixture was stirred overnight. The reaction was quenched by the dropwise addition of sat. NH_4_Cl solution (5 mL) and the organic solvent was removed. The aqueous layer was extracted with EtOAc (3 × 10 mL). The combined organic phases were dried over anhydrous Na_2_SO_4_, and concentrated under reduced pressure to give a residue. The crude product was purified by flash column chromatography (5–20% EtOAc/hexane) to give (*S*)-**9** as a white solid.

(*S*)-**9**: 86.3% yield (78 mg); [α]_D_^24^ = +13.9 (*c* 0.62, CH_2_Cl_2_); ^1^H NMR (400 MHz, CDCl_3_) δ 7.32–7.25 (m, 5H), 7.23–7.11 (m, 6H), 4.09–3.99 (m, 1H), 3.02 (d, *J* = 3.3 Hz, 1H), 2.89 (t, *J* = 7.5 Hz, 2H), 2.82–2.63 (m, 4H), 2.60–2.47 (m, 2H), 1.87–1.75 (m, 1H), 1.68 (ddd, *J* = 10.4, 7.1, 3.8 Hz, 1H); ^13^C NMR (100 MHz, CDCl_3_) δ 211.0, 141.8, 140.6, 128.6, 128.5, 128.4, 128.3, 126.2, 125.8, 66.8, 49.3, 45.0, 38.0, 31.7, 29.5; HRMS (EI): *m/z* [M]^+^ calcd for C_19_H_22_O_2_ 282.1620, observed 282.1615; HPLC: Chiralcel OJ-H column, IPA/hexane = 30/70, 1.0 mL/min, 210 nm; t_1_ = 10.5 [(*S*)-**9**], t_2_ = 12.9 min [(*R*)-**9**]; 99.7% *ee*.

Similarly, (*S*)-**11** [30] was prepared as above.

(*S*)-**11**: 83.1% yield (74 mg); [α]_D_^24^ = +27.5 (*c* 0.51, CHCl_3_); ^1^H NMR(400 MHz, CDCl_3_) δ 7.56 (dd, *J* = 5.2, 3.2 Hz, 2H), 7.47 (ddd, *J* = 6.6, 3.9, 1.3 Hz, 1H), 7.39 (t, *J* = 7.4 Hz, 2H), 7.29 (dd, *J* = 10.1, 4.6 Hz, 2H), 7.20 (dd, *J* = 14.0, 7.0 Hz, 3H), 4.21 (dd, *J* = 7.5, 3.9 Hz, 1H), 2.93–2.80 (m, 3H), 2.73 (dt, *J* = 13.6, 8.2 Hz, 2H), 1.95–1.84 (m, 1H), 1.77 (tdd, *J* = 11.2, 6.2, 3.5 Hz, 1H); ^13^C NMR (100 MHz, CDCl_3_) δ 187.3, 141.6, 133.1, 131.0, 128.6, 128.5, 128.5, 125.9, 119.6, 91.9, 87.8, 66.9, 52.3, 38.0, 31.7; HRMS (EI): *m*/*z* [M]^+^ calcd for C_19_H_18_O_2_ 278.1307, observed 278.1306; HPLC: Chiralcel OJ-H column, IPA/hexane = 30/70, 1.0 mL/min, 210 nm; t_1_ = 8.8 [(*S*)-**11**]; 99.9% *ee*.

## 4. Conclusions

We have developed a highly efficient and convenient method for the asymmetric total and formal syntheses of three natural products containing a 1,3-dioxygenated skeleton. Indeed, many linear diarylpentanoids and diarylheptanoids commonly share β-hydroxy ketone and 1,3-diol units. It was ambitioned that these natural products could be readily accessible from chiral 3-hydroxy-5-phenylpentanoic acid. Optically pure (3*S*)-hydroxy-5-phenylpentanoic acid was obtained from the diastereomeric Evans imides by chromatographic separation. This entailed an appealing strategy for the asymmetric synthesis of (*S*)-daphneolone, (*S*)-dihydroyashabushiketol, and the formal synthesis of (3*S*,5*S*)-yashabushidiol B, utilizing the Weinreb amide derived from the (*S*)-acid. The represented examples showed significant advantages over these previous synthetic approaches and the optical purity of natural products. More importantly, we believe this strategy provides a practical and convergent method enabling the rapid construction of these classes of molecules of interest. Further exploration to extend the scope of this protocol and their biological profiling is underway, and the results will be reported in due course.

## Data Availability

The data presented in this article are available in the Supplementary Materials.

## Supplementary Materials

The following supporting information can be downloaded at www.mdpi.com/article/10.3390/ijms26041476/s1.
