# Supplementary material for: Practical Preparation of (3S)-Hydroxy-5-Phenylpentanoic Acid: Asymmetric Synthesis of (S)-Daphneolone and (S)-Dihydroyashabushiketol, and Formal Synthesis of (3S,5S)-Yashabushidiol B
Source: Int J Mol Sci. 2025 Feb 10;26(4):1476. doi: 10.3390/ijms26041476 (PMC11854942; doi:10.3390/ijms26041476)
Supplement: Supplementary file 1 [file ijms-26-01476-s001.zip › ijms-3382582-supplementary.pdf]

Supporting Information

Practical preparation of (3*S*)-hydroxy-5-phenylpentanoic acid: Asymmetric synthesis of (*S*)-daphneolone, (*S*)-dihydroyashabushiketol, and formal synthesis of (3*S*,5*S*)-yashabushidiol B

So-Yeon Nam, Joungmo Cho, Simon MoonGeun Jung,* Hyun-Jun Lee, Hyung Won Ryu, Sei-Ryang Oh, and Kee-In Lee*

***Corresponding Authors**

Simon MoonGeun Jung: School of Food Biotechnology and Chemical Engineering, Hankyong National University, Anseong 17579, Republic of Korea; orcid.org/0000-0001-8269-8833; Email: mgjung@hknu.ac.kr

Kee-In Lee: Green Chemistry Division, Korea Research Institute of Chemical Technology, Daejeon 34114, Republic of Korea; Research and Development Center, Molecules & Materials Co., Ltd., Daejeon 34013, Republic of Korea; orcid.org/0000-0002-1341-0514; Email: kilee@krict.re.kr

General

Unless otherwise specified, all reactions were carried out under a nitrogen atmosphere in oven-dried glassware with magnetic stirring. All reagents and anhydrous solvents were purchased and used without any further purification. Thin layer chromatography (TLC) was performed using Merck aluminum foil-backed sheets pre-coated with Kieselgel 60F254. Column chromatography refers to chromatography on Merck Silica gel C60 (40–60 μm). The yield refers to isolated yield.

Melting points were determined in a capillary and were uncorrected. NMR spectra were recorded on a Bruker DPX-400, a Bruker AVANCE AV400, a Bruker DPX-500, or a Bruker AMX-500 spectrometer. The experiments were performed at 400 MHz for ^{1}H and 100 MHz for ^{13}C, except where otherwise specified. Chemical shifts (δ) are reported in parts per million (ppm) from tetramethylsilane with the undeuterated solvent resonance as the internal standard. Mass spectra (*m/z*) were recorded on a Waters ACQUITY UPLC H-Class / SQD2 Mass Spectrometer in Electrospray Ionization (ESI) or Atmospheric Pressure Chemical Ionization (APCI), and HRMS were recorded on a JEOL JMS-700 in Chemical Ionization (CI), Electron Impact (EI), or Fast atom bombardment (FAB) modes. Optical rotations were determined on a Perkin-Elmer 241 polarimeter in a 1 dm cell. Concentrations are given in g/100 mL. The determination of values of enantiomeric excess (*ee*) and diastereomeric excess (*de*) was obtained by high-performance liquid chromatography (HPLC) using Thermo/Dionex UltiMate 3000 HPLC System equipped with Chiralcel OJ-H column (4.6 x 250 mm), and analytical conditions were specified.

Preliminary Result

1) Aldol addition of (*S*)-3-acetyl-4-isopropyl-2-oxazolidinone with 3-phenylpropanal

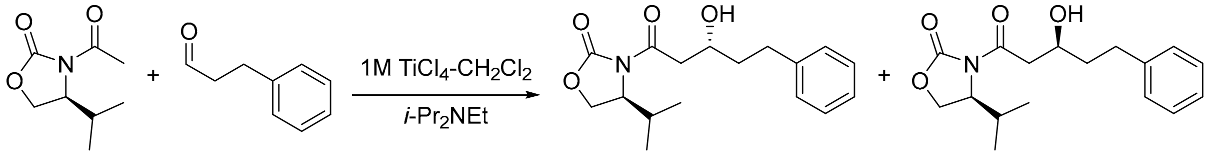

(3'*R*,4*S*)-**2** (3'*S*,4*S*)-**2**

Aldol condensation of (*S*)-3-acetyl-4-isopropyl-2-oxazolidinone and 3-phenylpropanal with $TiCl_4$ (1.0 M in CH_2Cl_2) and *i*-Pr_2NEt gave two diastereomers and HPLC analysis shows (3'*R*,4*S*)-**2**/ (3'*S*,4*S*)-**2** = 5.6/1. These were readily separated by silica gel column chromatography. Their stereochemistry was judged by the diastereotopic methylene protons adjacent to the imide carbonyl as observed previously.[1] However, the desired product, (3'*S*,4*S*)-**2** was a minor amount. HPLC: Chiralcel OJ-H column, IPA/hexane = 30/70, 1.0 mL/min, 210 nm; t_1 = 11.6 [(3'*R*,4*S*)-**2**], t_2 = 13.3 min [(3'*S*,4*S*)-**2**].

HPLC chromatogram of the reaction mixture

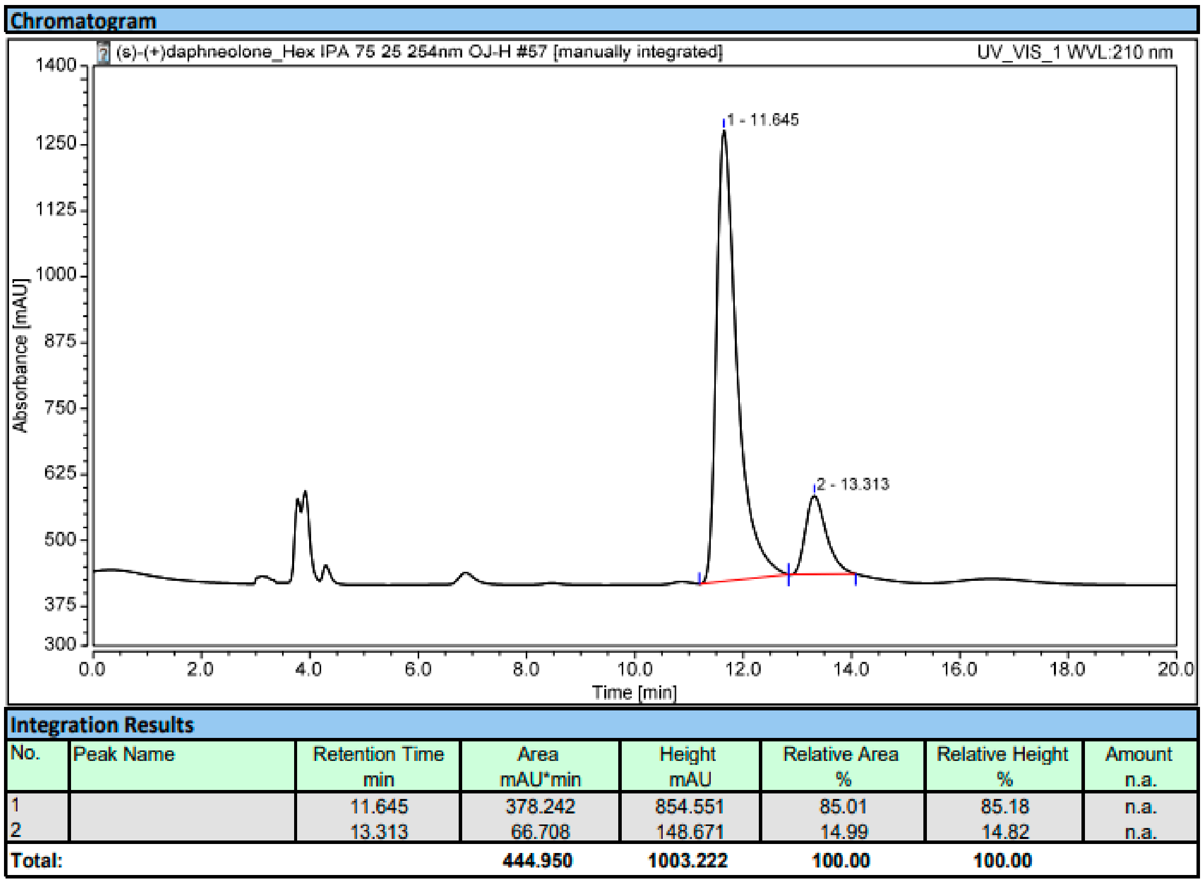

Integration Results

No.	Peak Name	Retention Time min	Area mAU*min	Height mAU	Relative Area %	Relative Height %	Amount n.a.
1		11.645	378.242	854.551	85.01	85.18	n.a.
2		13.313	66.708	148.671	14.99	14.82	n.a.
**Total:**			**444.950**	**1003.222**	**100.00**	**100.00**	

I. Synthesis of (*S*)-3-hydroxy-5-phenylpentanoic acid

1. Preparation of (*R*)-3-acetyl-4-isopropyl-2-oxazolidinone, (*R*)-**1**

According to the previous procedures, (*R*)-**1** was prepared from (*R*)-4-isopropyl-2-oxazolidinone and acetyl chloride with sodium hydride.[2]

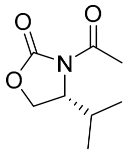

(*R*)-**1:** 92.8% yield (6.35 g); ^{1}H NMR (400 MHz, $CDCl_3$) δ 4.48-4.37 (m, 1H), 4.31-4.17 (m, 2H), 2.54 (s, 3H), 2.45-2.35 (m, 1H), 0.92 (d, *J* = 7.0 Hz, 3H), 0.88 (d, *J* = 6.9 Hz, 3H); ^{13}C NMR (100 MHz, $CDCl_3$): δ 170.2, 154.3, 63.3, 58.3, 28.3, 23.8, 17.9, 14.6.

2. Aldol addition of (*R*)-**1** with 3-phenylpropanal

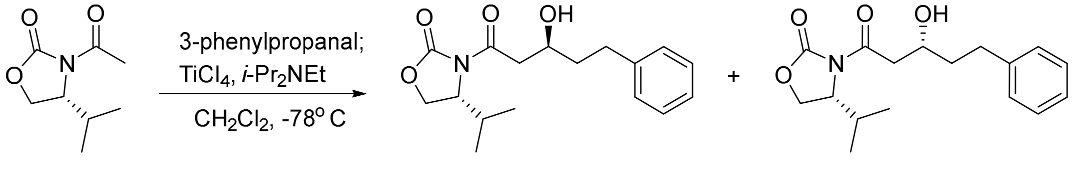

(3'*S*,4*R*)-**2** (3'*R*,4*R*)-**2**

To a stirred solution of (*R*)-**1** (5.0 g, 29.2 mmol) in methylene chloride (150 mL) at -78 ˚C under nitrogen, 1.0 M titanium(IV) chloride in methylene chloride (58.4 mL, 58.4 mmol) was added. After 10 min, diisopropylethylamine (9.93 mL, 58.4 mmol) was added and stirred for 1 h at the same temperature, followed by 3-phenylpropanal (7.68 mL, 58.4 mmol). The reaction mixture was maintained at -78 ˚C for 5 h and warmed to room temperature overnight, and then a saturated ammonium chloride solution (50 mL) was added. The organic layer was washed with water (50 mL) and then brine (50 mL), dried over anhydrous magnesium sulfate and concentrated *in vacuo* to give the diastereomers. They were separated by silica gel chromatography eluting with 15% EtOAc/petroleum ether to give (3'*S*,4*R*)-**2** as the major and **(**3'*R*,4*R*)-**2**, respectively in 58.2% overall yield.

(3'*S*,4*R*)-**2:** 48.9% yield (4.36 g); 99.8% *de*; ^{1}H NMR (400 MHz, $CDCl_3$) δ 7.32-7.26 (m, 2H), 7.24-7.11 (m, 3H), 4.49-4.39 (m, 1H), 4.33-4.18 (m, 2H), 4.07 (dt, *J* = 9.8, 5.9 Hz, 1H), 3.11 (d, *J* = 6.1 Hz, 2H), 3.05 (d. *J* = 4.4 Hz, 1H), 2.89-2.79 (m, 1H), 2.73 (ddd. *J* = 13.8, 9.2, 7.1 Hz, 1H), 2.45-2.31 (m, 1H), 1.97-1.86 (m, 1H), 1.85-1.73 (m, 1H), 0.93 (d. *J* = 7.0 Hz, 3H), 0.88 (d, *J* = 6.9 Hz, 3H); ^{13}C NMR (100 MHz, $CDCl_3$): δ 172.7, 154.1, 141.7, 128.5, 128.4,

125.8, 67.2, 63.5, 58.4, 42.5, 38.1, 31.7, 28.4, 17.9, 14.7; HRMS (EI): *m/z* $[M]^+$ calcd for $C_{17}H_{23}NO_4$ 305.1627, observed 305.1628; HPLC: Chiralcel OJ-H column, IPA/hexane = 30/70, 1.0 mL/min, 210 nm; t_1 = 12.7 [(3'*R*,4*R*)-**2**], t_2 = 13.9 min [(3'*S*,4*R*)-**2**].

HPLC chromatogram of (3'*S*,4*R*)-**2**

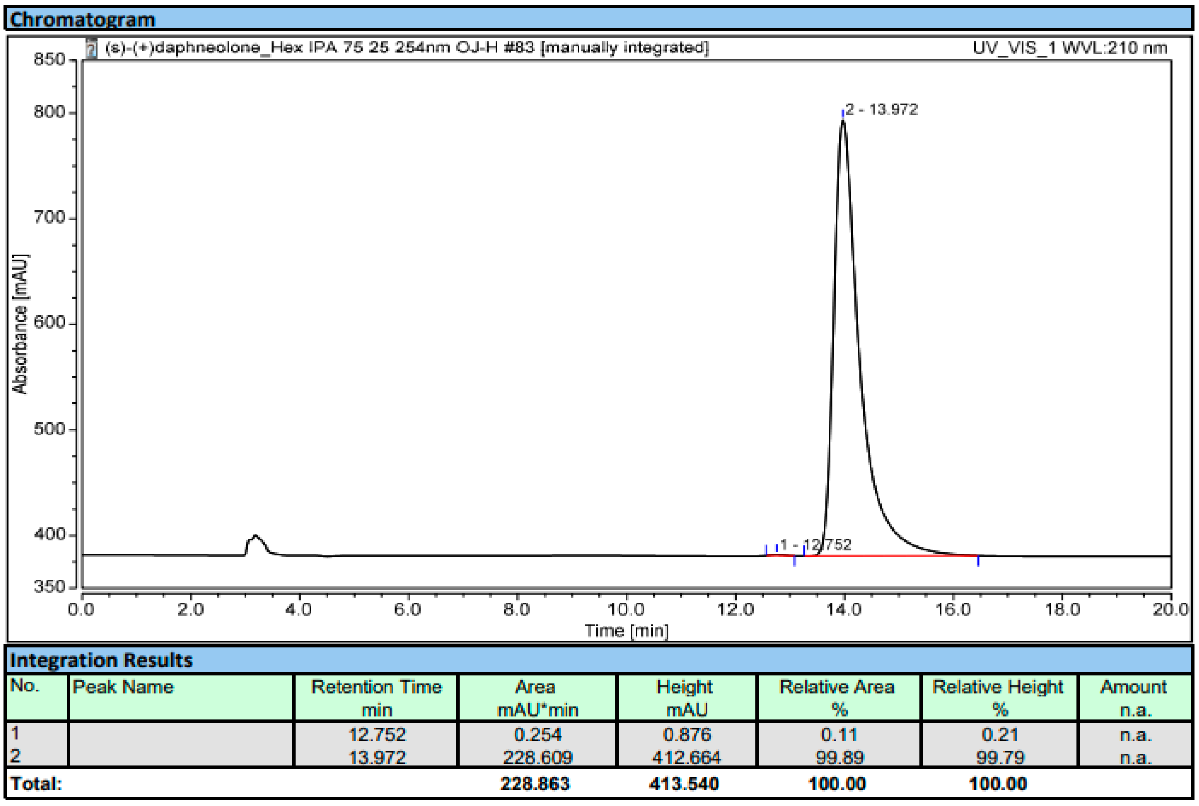

Integration Results

No.	Peak Name	Retention Time min	Area mAU*min	Height mAU	Relative Area %	Relative Height %	Amount n.a.
1		12.752	0.254	0.876	0.11	0.21	n.a.
2		13.972	228.609	412.664	99.89	99.79	n.a.
**Total:**			**228.863**	**413.540**	**100.00**	**100.00**	

(3’*R*,4*R*)-**2:** 9.3% yield (0.83 g); 92.4% *de*; ^{1}H NMR (400 MHz, $CDCl_3$): δ 7.28 (dd, *J* = 12.6, 5.2 Hz, 2H), 7.20 (dd, *J* = 15.8, 7.3 Hz, 3H), 4.49-4.39 (m, 1H), 4.34-4.19 (m, 2H), 4.16-4.05 (m, 1H), 3.20 (dd, *J* = 17.5, 2.6 Hz, 1H), 2.99 (dd, *J* = 17.5, 9.2 Hz, 1H), 2.95 (d, *J* = 4.7 Hz, 1H), 2.89-2.79 (m, 1H), 2.73 (ddd, *J* = 13.8, 9.2, 7.1 Hz, 1H), 2.42-2.30 (m, 1H), 1.96-1.85 (m, 1H), 1.85-1.73 (m, 1H), 0.92 (d, *J* = 7.0 Hz, 3H), 0.88 (d, *J* = 6.9 Hz, 3H); ^{13}C NMR (100 MHz, $CDCl_3$): δ 172.7, 154.0, 141.7, 128.5, 128.4, 125.8, 67.1, 63.5, 58.4, 42.6, 38.0, 31.7, 28.4, 17.9, 14.7; HRMS (EI): *m/z* $[M]^+$ calcd for $C_{17}H_{23}NO_4$ 305.1627, observed 305.1630; HPLC: Chiralcel OJ-H column, IPA/hexane = 30/70, 1.0 mL/min, 210 nm; t_1 = 12.6 [(3’*R*,4*R*)-**2**], t_2 = 13.8 min [(3’*S*,4*R*)-**2**].

HPLC chromatogram of (3’*R*,4*R*)-**2**

Chromatogram

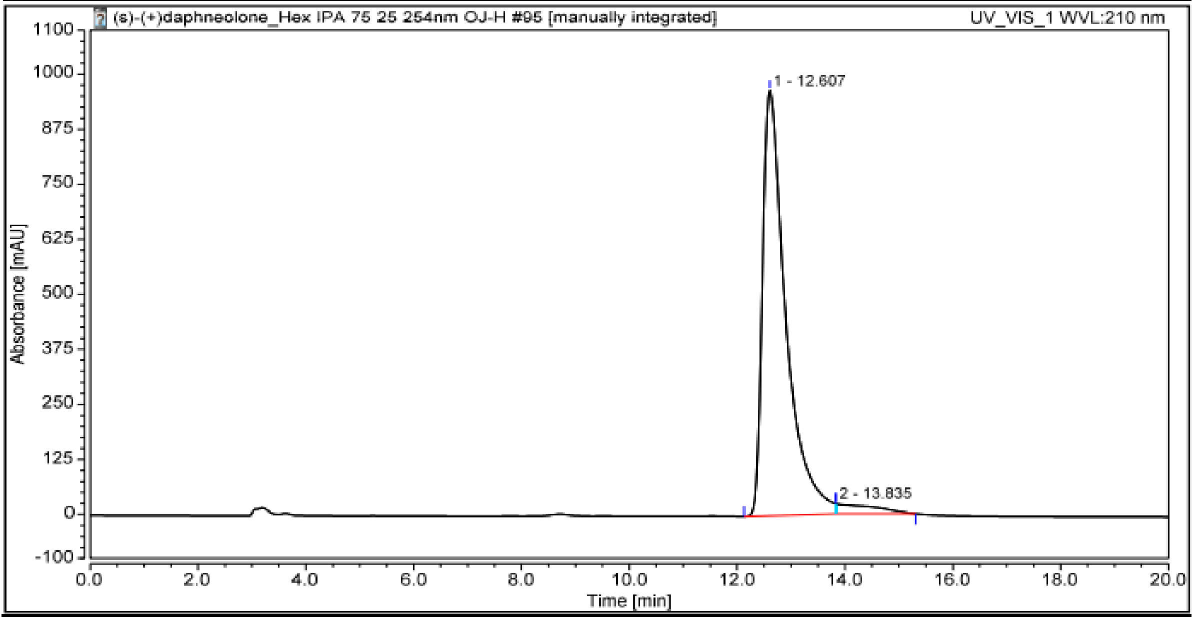

Integration Results

No.	Peak Name	Retention Time min	Area mAU*min	Height mAU	Relative Area %	Relative Height %	Amount n.a.
1		12.607	483.134	965.311	96.21	97.66	n.a.
2		13.835	19.043	23.144	3.79	2.34	n.a.
**Total:**			**502.177**	**988.456**	**100.00**	**100.00**	

3. Synthesis of (*S*)-3-hydroxy-5-phenylpentanoic acid, (*S*)-**3**[3]

A solution of the imide (3'*S*,4*R*)-**2** (2.50 g, 8.18 mmol) in THF (80 mL) and water (20 mL) was treated with LiOH (310 mg, 12.9 mmol) and 30 wt% H_2O_2 in water (3.80 mL, 37.2 mmol) at 0 °C. After stirring at the same temperature for 6 h, the reaction was quenched by adding sat. $NaHCO_3$ solution (50 mL) and the organic solvent was removed. The aqueous layer was washed with CH_2Cl_2 (3 x 20 mL), acidified with 1N HCl adjusted to pH = 2, and extracted with Et_2O (3 x 30 mL). The combined organic phases were dried over anhydrous Na_2SO_4, and concentrated under reduced pressure to give (*S*)-**3** as a white solid.

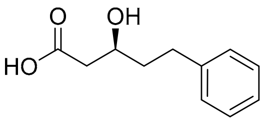

(*S*)-**3:** 88.7% (1.41 g); mp = 134-126 °C; $[\alpha]_D^{24}$ = -43.4 (*c* 1.06, MeOH) [lit.[3] $[\alpha]_D^{25.4}$ = -42.68 (*c* 1.3, MeOH)]; ^{1}H NMR (400 MHz, DMSO-d_6): δ 7.28 (t, *J* = 7.4 Hz, 2H), 7.23-7.08 (m, 3H), 3.82 (m, 1H), 3.35 (br s, 1H), 2.51 (m, 1H), 2.37 (m, 1H), 2.36 (dd, *J* = 14.8, 5.0 Hz, 1H), 2.27 (dd, *J* = 14.8, 8.0 Hz, 1H), 1.67-1.63 (m, 2H); ^{13}C NMR (100 MHz, DMSO-d_6): δ 173.4, 142.6, 128.8, 128.7, 126.0, 67.0, 43.1, 39.2, 31.7; MS (ESI): *m/z* 194.1 $[M]^+$.

The optical purity of (*S*)-**3** was determined after derivatization to its corresponding methyl ester (MeI, Cs_2CO_3, DMF; 87%) and shows 98.5% *ee*.

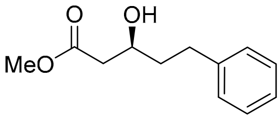

(*S*)-**3**-OMe

HPLC: Chiralcel OJ-H column, IPA/hexane = 30/70, 1.0 mL/min, 210 nm; t_1 = 5.5 [(*S*)-**3**-OMe], t_2 = 8.2 min [(*R*)-**3**-OMe].

HPLC chromatogram of methyl (*S*)-3-hydroxy-5-phenylpentanoate, (*S*)-**3**-OMe

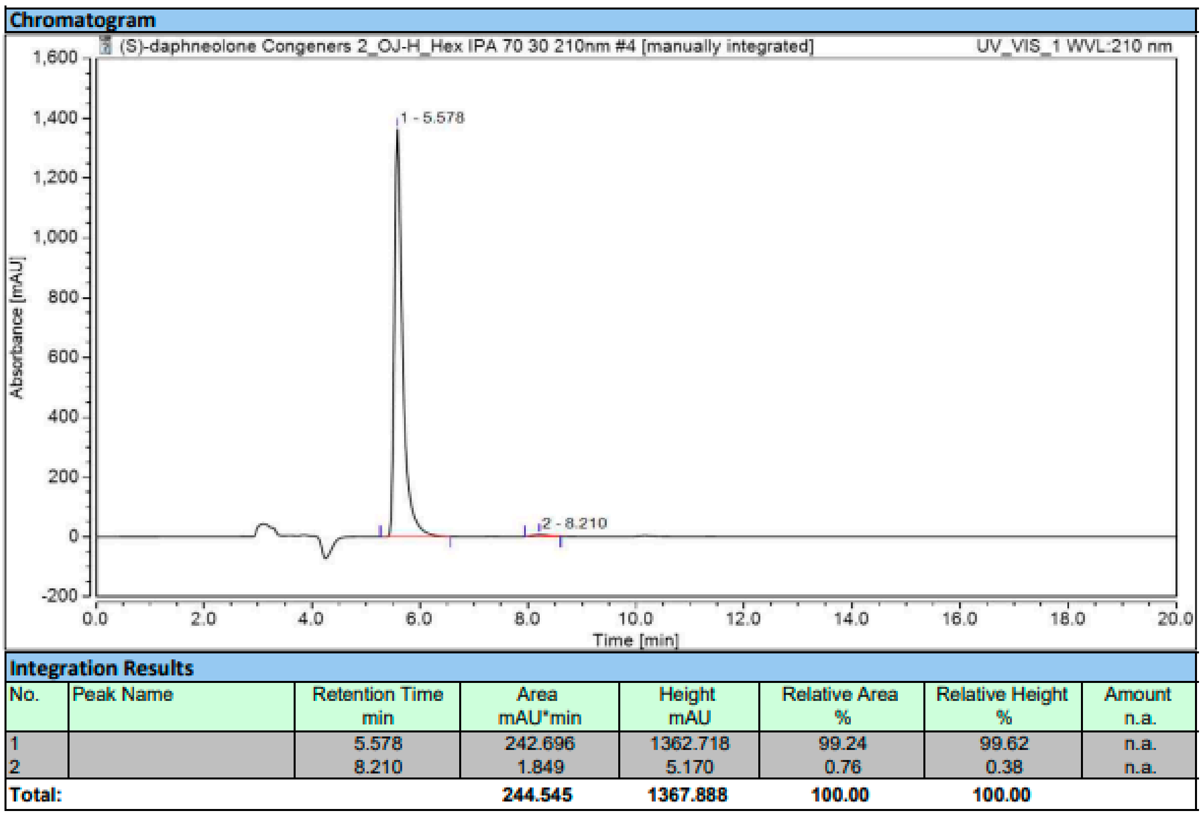

Integration Results

No.	Peak Name	Retention Time min	Area mAU*min	Height mAU	Relative Area %	Relative Height %	Amount n.a.
1		5.578	242.696	1362.718	99.24	99.62	n.a.
2		8.210	1.849	5.170	0.76	0.38	n.a.
**Total:**			**244.545**	**1367.888**	**100.00**	**100.00**	

4. Synthesis of Weinreb amide, (*S*)-**4**

To a solution of (*S*)-**3** (300 mg, 1.54 mmol) in DMF (6 mL) was treated with EDC-HCl (445 mg, 2.32 mmol), HOBt (313 mg, 2.32 mmol), MeONHMe-HCl (313 mg, 2.32 mmol), and Et_3N (0.64 mL, 4.62 mmol). The reaction was stirred at room temperature overnight. After completion, the reaction mixture was diluted with EtOAc (50 mL), and the combined were successively washed with sat. $NaHCO_3$ solution, water, and then brine. The organic layer was dried over anhydrous MgSO4 and concentrated *in vacuo*. The crude was purified by silica gel column chromatography (5% MeOH/CH_2Cl_2) to provide (*S*)-**4** as a colorless oil.

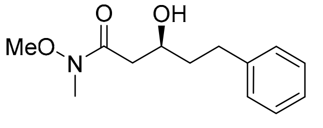

(*S*)-**4**[4]**:** 83.2% (304 mg)**;** $[\alpha]_D^{24}$ = +33.6 (*c* 0.52, $CHCl_3$) [lit. $[\alpha]_D^{22}$ = +34.5 (*c* 0.5, $CHCl_3$)[4a] and $[\alpha]_D^{25}$ = +28.6 (*c* 1.08, $CHCl_3$)[4b]]**;** ^{1}H NMR (400 MHz, $CDCl_3$): δ 7.29-7.15 (m, 5H), 4.03 (m, 1H), 3.86 (d, *J* = 2.6 Hz, 1H), 3.66 (s, 3H), 3.18 (s, 3H), 2.83 (td, *J* = 9.3, 5.0 Hz, 1H), 2.74-2.63 (m, 1H), 2.51 (dd, *J* = 14.6, 5.8 Hz, 1H), 1.87 (td, *J* = 9.6, 4.4 Hz, 1H), 1.76 (m, 1H); ^{13}C NMR (100 MHz, $CDCl_3$): δ 173.8, 142.0, 128.5, 128.3, 125.8, 67.2, 61.2, 38.2 (2C), 31.8 (2C); HRMS (EI): *m/z* $[M]^+$ calcd for $C_{13}H_{19}NO_3$ 237.1365, observed 237.1366.

5. Preparation of TBS ether, (*S*)-**5**

To a stirred solution of (*S*)-**4** (2.40 g, 10.1 mmol) in DMF (30 mL) was added TBSCl (1.83 g, 12.1 mmol) and imidazole (1.03 g, 15.2 mmol. The reaction mixture was stirred at room temperature overnight and was quenched with sat. NH_4Cl solution (150 mL). The resulting was extracted with EtOAc (3 x 50 mL) and the combined were washed with water (50 mL) and brine (50 mL), dried over anhydrous $MgSO_4$ and concentrated *in vacuo*. The crude was purified by silica gel column chromatography (EtOAc/Hex = 1/9) to provide (*S*)-**5** as a colorless oil.

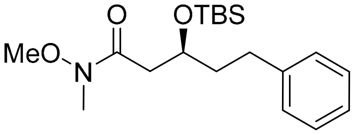

(*S*)-**5:** 89.5% (3.18 g); ^{1}H NMR (400 MHz, $CDCl_3$): δ 7.29-7.15 (m, 5H), 4.31 (quintet, *J* = 6.4 Hz, 1H), 3.68 (s, 3H), 3.17 (s, 3H), 2.73-2.64 (m, 3H), 2.46 (dd, *J* = 14.8, 5.6 Hz, 1H), 1.87-1.77 (m, 2H), 0.89 (s, 9H), 0.08 (s, 3H), 0.04 (s, 3H); ^{13}C NMR (100 MHz, $CDCl_3$): δ 172.3, 142.4, 128.3, 127.7, 69.1, 61.3, 39.7, 39.6, 31.9, 31.5, 25.9, 18.0, -4.58, -4.69; MS (ESI): *m/z* 374.2 [M + Na]$^+$.

II. Synthesis of (*S*)-Daphneolone

1. Preparation of *rac*-daphneolone

According to literature procedures, *rac*-daphneolone was prepared from 4-TMSO-PhCOMe with 3-phenylpropanal through aldol addition (LDA, -78 ºC, THF).[5]

***rac*-Daphneolone.** HPLC: Chiralcel OJ-H column, IPA/hexane = 25/75, 1.0 mL/min, 254 nm; t_1 = 10.2 (*S*), t_2 = 12.5 min (*R*).

HPLC chromatogram of *rac*-daphneolone

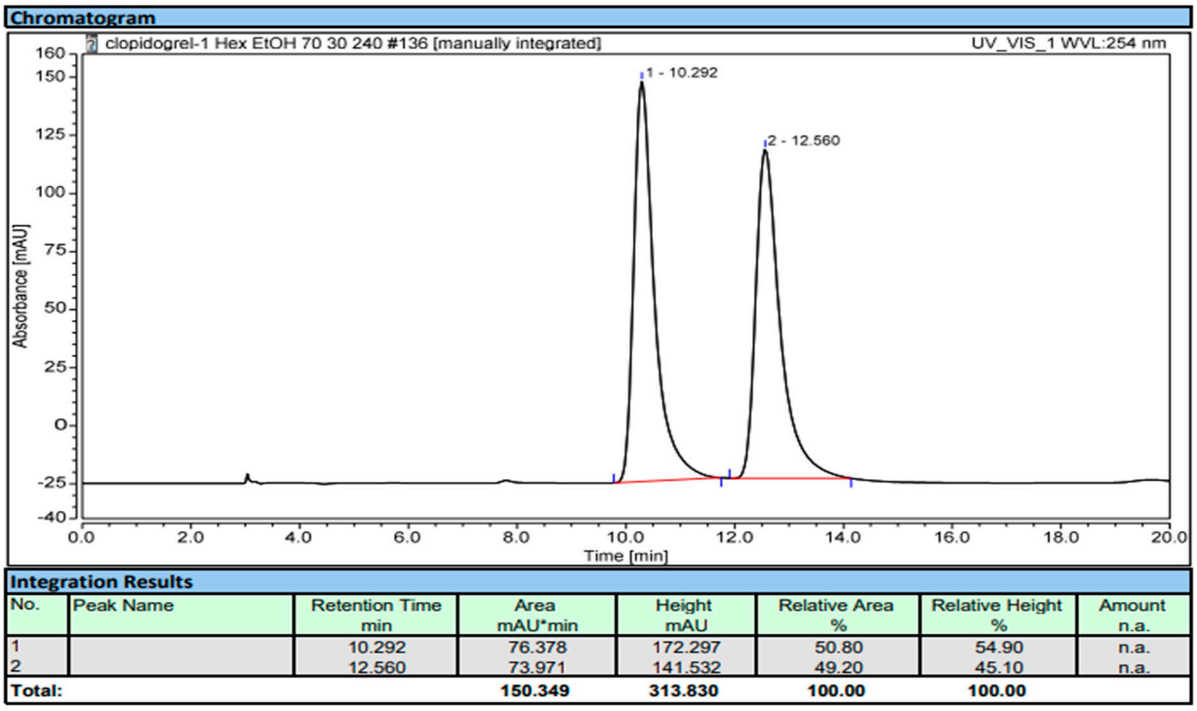

Integration Results

No.	Peak Name	Retention Time min	Area mAU*min	Height mAU	Relative Area %	Relative Height %	Amount n.a.
1		10.292	76.378	172.297	50.80	54.90	n.a.
2		12.560	73.971	141.532	49.20	45.10	n.a.
**Total:**			**150.349**	**313.830**	**100.00**	**100.00**	

2. Isolation of (*S*)-daphneolone

Natural daphneolone was isolated from *Daphne giraldii* callus according to the literature procedures and identified as (*S*)-(+)-daphneolone.[6]

HPLC chromatogram of natural (*S*)-daphneolone

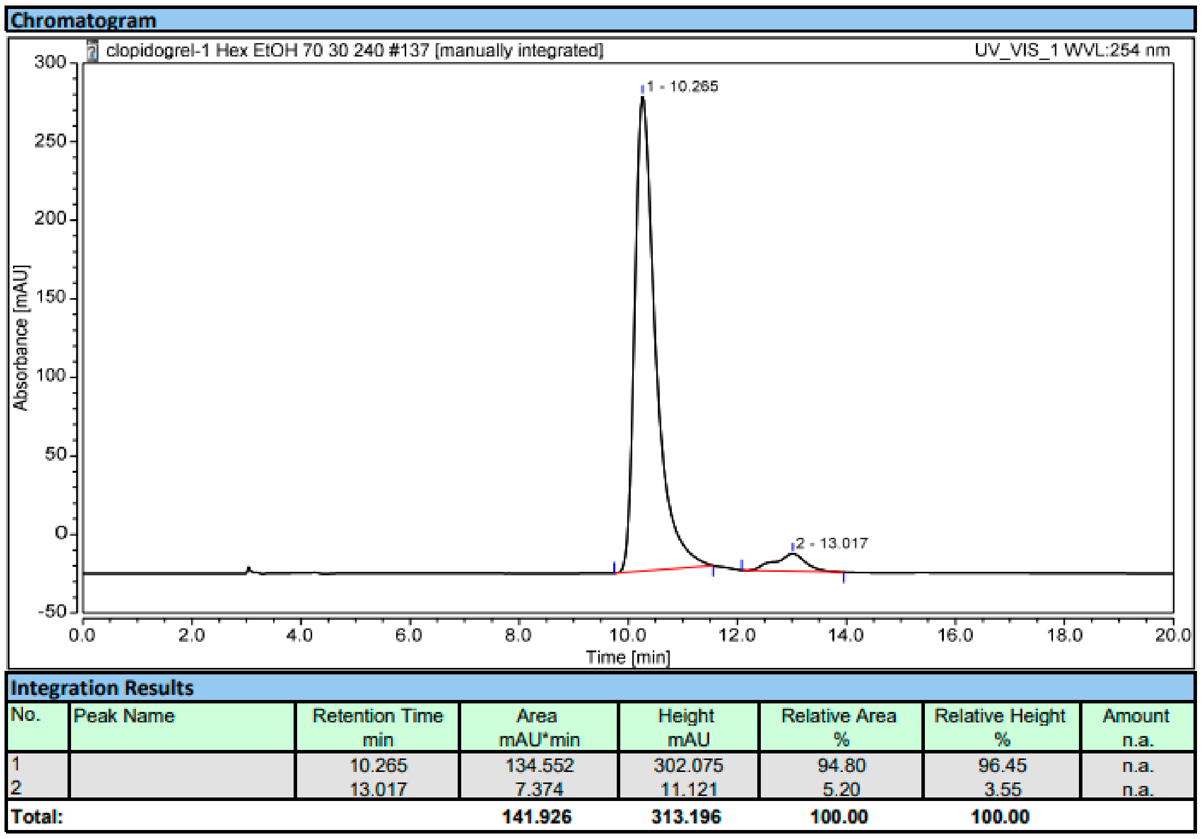

Integration Results

No.	Peak Name	Retention Time min	Area mAU*min	Height mAU	Relative Area %	Relative Height %	Amount n.a.
1		10.265	134.552	302.075	94.80	96.45	n.a.
2		13.017	7.374	11.121	5.20	3.55	n.a.
**Total:**			**141.926**	**313.196**	**100.00**	**100.00**	

2. Synthesis of (*S*)-daphneolone

2-1. Synthesis of β-hydroxyketone, (*S*)-**6**

To a stirred solution of 4-Br-PhOTBS (1.94 g, 6.75 mmol) in THF (8 mL) at -78 °C under nitrogen was added dropwise 1.7M *t*-BuLi in hexane (7.7 mL, 13.1 mmol). The solution was stirred at the same temperature for 30 min, then placed at room temperature, and stirred for 30 min. To this solution cooled again to -78 °C was added dropwise (*S*)-**5** (766 mg, 2.18 mmol) in THF (7 mL). The resulting was kept at -78 °C stirring for over 2 h, when poured into sat. NH_4Cl solution (25 mL). The mixture was diluted with 20% EtOAc/hexane (100 mL) and the layers were separated. The aqueous layer was extracted with 20% EtOAc/hexane (3 × 30 mL), the combined organic layers were washed successively with water (30 mL) and brine (30 mL), dried over anhydrous $MgSO_4$, and concentrated *in vacuo*. The crude was purified by silica gel column chromatography (2% EtOAc/hexane) to provide (*S*)-**6** as a colorless oil.

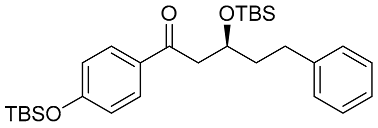

(*S*)-**6:** 42.9% (467 mg); ^{1}H NMR (400 MHz, $CDCl_3$): δ 7.94 (d, *J* = 8.7 Hz, 2H), 7.36-7.24 (m, 5H), 6.93 (d, *J* = 8.6 Hz, 2H), 4.49 (quintet, *J* = 6.2 Hz, 1H), 3.28 (dd, *J* = 15.2, 6.8 Hz, 1H), 2.99 (dd, *J* = 15.2, 5.6 Hz, 1H), 2.80-2.77 (m, 2H), 1.97-1.90 (m, 2H), 1.06 (s, 9H), 0.91 (s, 9H), 0.30 (s, 6H), 0.13 (s, 3H), 0.07 (s, 3H); ^{13}C NMR (100 MHz, $CDCl_3$): δ 197.8, 160.2, 142.3, 131.3, 130.6, 128.4, 128.3, 125.7, 119.8, 69.4, 45.6, 31.4, 25.8, 25.6, 18.2, 18.0, -4.36, -4.60, -4.67; MS (ESI): *m/z* 521.3 $[M + Na]^+$.

2-2. Synthesis of (*S*)-daphneolone, (*S*)-**7**

To a cold (0 ºC) solution of (*S*)-**6** (270 mg, 0.54 mmol) in anhydrous THF (10 mL), was added 1M TBAF in THF (2.16 mL, 2.16 mmol), and the mixture was stirred overnight allowing the mixture to warm to room temperature. the reaction was quenched by the dropwise addition of sat. NH_4Cl solution (5 mL) and the organic solvent was removed. The aqueous layer was extracted with EtOAc (3 x 10 mL). The combined organic phases were dried over anhydrous Na_2SO_4, and concentrated under reduced pressure to give a residue. The crude product was purified by flash column chromatography (5-20% EtOAc/hexane) to give (*S*)-**7** as a white solid.

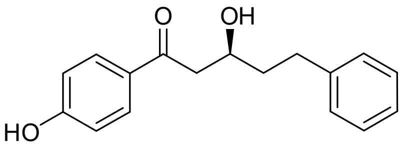

(*S*)-**7**: 82.2% (120 mg); mp 118-120 ºC; $[\alpha]_D^{24}$ = +5.7 (*c* 0.16, MeOH) [lit. $[\alpha]_D^{23}$ = +10 (*c* 1.1, MeOH)[6a] and $[\alpha]_D^{17}$ = +5.3 (*c* 0.13, MeOH)[6b]]; 99.9% *ee*; ^1H NMR (400 MHz, CD_3OD): δ 7.86 (d, *J* = 8.8 Hz, 2H), 7.24-7.13 (m, 5H), 6.82 (d, *J* = 8.8 Hz, 2H), 4.16 (septet, *J* = 4.6 Hz, 1H), 3.12 (dd, *J* = 15.8, 7.9 Hz, 1H), 3.00 (dd, *J* = 15.8, 4.5 Hz, 1H), 2.76 (m, 1H), 2.62 (m, 1H), 1.84-1.80 (m, 2H); ^{13}C NMR (100 MHz, CD_3OD): δ 198.6, 162.5, 142.0, 130.6, 129.0, 128.0, 127.9, 125.3, 114.8, 67.5, 45.1, 38.8, 31.5; HRMS (EI): *m/z* $[M]^+$ calcd for $C_{17}H_{18}O_3$ 270.1256, observed 270.1250.

HPLC chromatogram of (*S*)-daphneolone

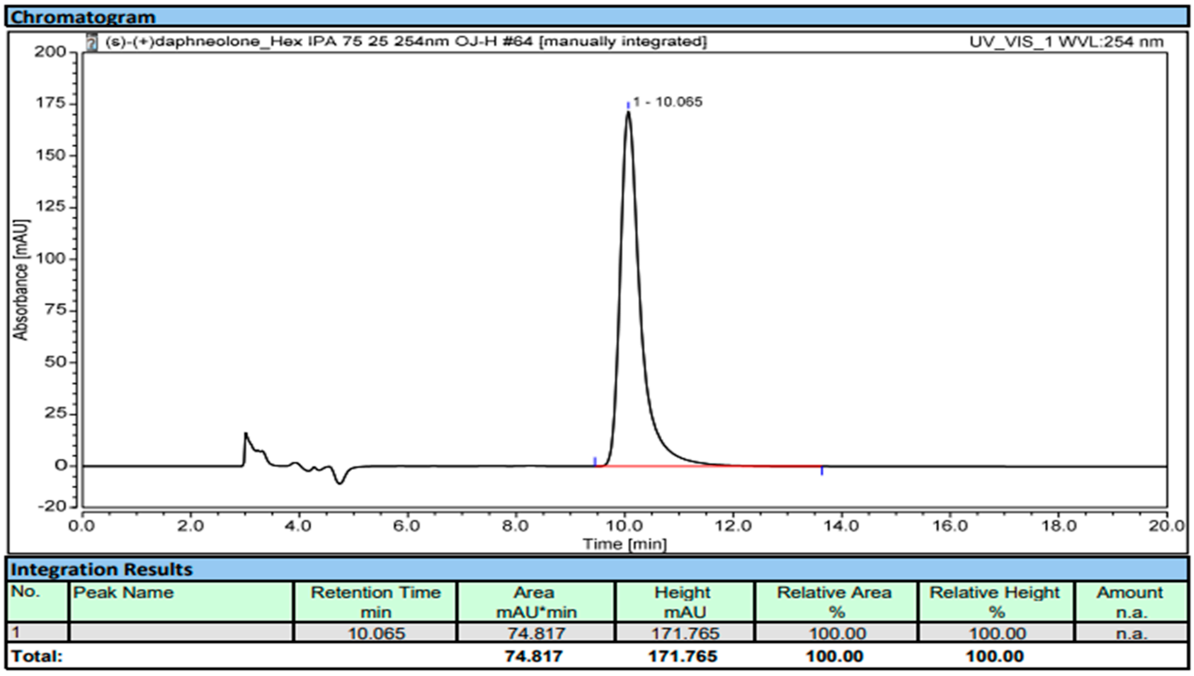

Integration Results

No.	Peak Name	Retention Time min	Area mAU*min	Height mAU	Relative Area %	Relative Height %	Amount n.a.
1		10.065	74.817	171.765	100.00	100.00	n.a.
**Total:**			**74.817**	**171.765**	**100.00**	**100.00**	

III. Synthesis of (*S*)-dihydroyashabushiketol and formal synthesis of (3*S*,5*S*)-yashabushidiol

1. Typical procedure for the synthesis of (*S*)-**10**

To a stirred solution of (*S*)-**5** (300 mg, 0.85 mmol) in THF (3 mL) at -78 °C under nitrogen was added dropwise 2.56 mL of phenylethynylmagnesium bromide (1.0 M in THF). The solution was stirred at -78 °C for 30 min, and then placed at room temperature overnight. The solution was poured into sat. NH_4Cl solution (25 mL) and the mixture was diluted with 20% EtOAc/hexane (30 mL) and the layers were separated. The aqueous layer was extracted with 20% EtOAc/hexane (3 × 10 mL), the combined organic layers were washed successively with water (30 mL) and brine (30 mL), dried over anhydrous $MgSO_4$, and concentrated *in vacuo*. The crude was purified by silica gel column chromatography (2-5% EtOAc/hexane) to provide (*S*)-**10** as a colorless oil.

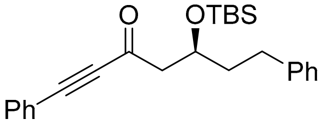

(*S*)-10: 79.4% yield (265 mg); ^{1}H NMR (400 MHz, $CDCl_3$) δ 7.55 (dd, *J* = 8.3, 1.3 Hz, 2H), 7.45 (d, *J* = 7.5 Hz, 1H), 7.39 (dd, *J* = 8.0, 6.7 Hz, 2H), 7.30-7.25 (m, 3H), 7.19 (d, *J* = 7.5 Hz, 3H), 4.41 (dd, *J* = 6.8, 5.6 Hz, 1H), 2.92 (dd, *J* = 14.9, 7.0 Hz, 1H), 2.82 (dd, *J* = 14.9, 5.6 Hz, 1H), 2.75-2.65 (m, 2H), 1.89 (ddt, *J* = 11.3, 9.0, 5.6 Hz, 2H), 0.93-0.85 (m, 9H), 0.12-0.03 (m, 6H); ^{13}C NMR (100 MHz, $CDCl_3$) δ 186.0, 141.9, 133.0, 130.7, 128.6, 128.4, 128.3, 125.8, 119.9, 91.1, 88.4, 68.6, 53.1, 39.4, 31.3, 25.9, 18.1, -4.4, -4.6; MS (ESI): *m/z* 415.2 $(M + Na)^+$.

Similarly, (*S*)-**8** was prepared using phenethylmagnesium chloride (1.0 M in THF).

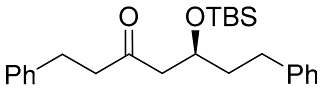

(*S*)-**8:** 93.1% yield (314 mg); ^{1}H NMR (400 MHz, $CDCl_3$) δ 7.27 (dd. *J* = 7.4, 6.0 Hz, 5H), 7.17 (dt, *J* = 12.8, 4.4 Hz, 6H), 4.24 (dd, *J* = 6.7, 5.6 Hz, 1H), 2.92-2.84 (m, 2H), 2.74 (dd, *J* = 11.2, 4.6 Hz, 2H), 2.64 (ddd, *J* = 12.9, 9.8, 5.2 Hz, 3H), 2.49 (dd, *J* = 15.3, 5.3 Hz, 1H), 1.76 (ddd, *J* = 8.3, 7.2, 4.3 Hz, 2H), 0.88 (s, 9H), 0.07 (s, 3H), 0.01 (s, 3H); ^{13}C NMR (100 MHz, $CDCl_3$) δ 208.7, 142.1, 141.0, 128.5, 128.4, 128.3, 126.0, 125.8, 68.7, 50.1, 46.1, 39.4, 31.4, 29.5, 25.9, 18.0, -4.5, -4.7; MS (ESI): *m/z* 419.3 $(M + Na)^+$.

2. Typical procedure for the synthesis of (*S*)-dihydroyashabushiketol, (*S*)-**9**

To a solution of (*S*)-**8** (127 mg, 0.32 mmol) in EtOH (10 mL), 1N HCl in water (7 mL) and the mixture was stirred overnight. The reaction was quenched by the dropwise addition of sat. NH_4Cl solution (5 mL) and the organic solvent was removed. The aqueous layer was extracted with EtOAc (3 x 10 mL). The combined organic phases were dried over anhydrous Na_2SO_4, and concentrated under reduced pressure to give a residue. The crude product was purified by flash column chromatography (5-20% EtOAc/hexane) to give (*S*)-**9** as a white solid.

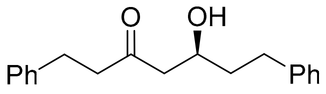

(*S*)-**9**[7]**:** 86.3% yield (78 mg); $[\alpha]_D^{24}$ = +13.9 (*c* 0.62, CH_2Cl_2) [lit.[7] $[\alpha]_D^{20}$ = +12.8 (*c* 1.16, CH_2Cl_2)]; ^{1}H NMR (400 MHz, $CDCl_3$) δ 7.32-7.25 (m, 5H), 7.23-7.11 (m, 6H), 4.09-3.99 (m, 1H), 3.02 (d, *J* = 3.3 Hz, 1H), 2.89 (t, *J* = 7.5 Hz, 2H), 2.82-2.63 (m, 4H), 2.60-2.47 (m, 2H), 1.87-1.75 (m, 1H), 1.68 (ddd, *J* = 10.4, 7.1, 3.8 Hz, 1H); ^{13}C NMR (100 MHz, $CDCl_3$) δ 211.0, 141.8, 140.6, 128.6, 128.5, 128.4, 128.3, 126.2, 125.8, 66.8, 49.3, 45.0, 38.0, 31.7, 29.5; HRMS (EI): *m/z* $[M]^+$ calcd for $C_{19}H_{22}O_2$ 282.1620, observed 282.1615; HPLC: Chiralcel OJ-H column, IPA/hexane = 30/70, 1.0 mL/min, 210 nm; t_1 = 10.5 [(*S*)-**9**], t_2 = 12.9 min [(*R*)-**9**]; 99.7% *ee*.

HPLC chromatogram of (*S*)-**9**

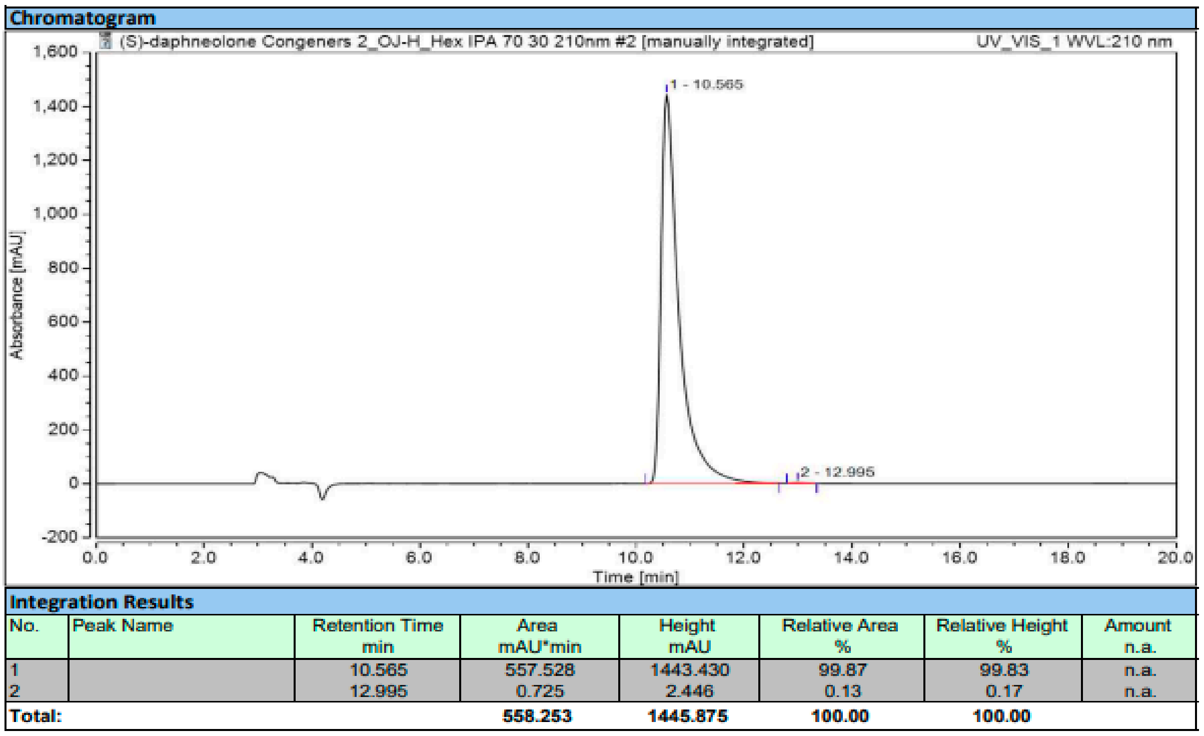

Integration Results

No.	Peak Name	Retention Time min	Area mAU*min	Height mAU	Relative Area %	Relative Height %	Amount n.a.
1		10.565	557.528	1443.430	99.87	99.83	n.a.
2		12.995	0.725	2.446	0.13	0.17	n.a.
**Total:**			**558.253**	**1445.875**	**100.00**	**100.00**	

Similarly, (S)-**11** was prepared as above.

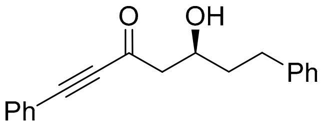

(S)-11[4a]: 83.1% yield (74 mg); $[\alpha]_D^{24}$ = +27.5 (c 0.51, $CHCl_3$) [lit.[4a] $[\alpha]_D^{26}$ = +27.6 (c 0.5, $CHCl_3$)]; ^{1}H NMR(400 MHz, $CDCl_3$) δ 7.56 (dd, J = 5.2, 3.2 Hz, 2H), 7.47 (ddd, J = 6.6, 3.9, 1.3 Hz, 1H), 7.39 (t, J = 7.4 Hz, 2H), 7.29 (dd, J = 10.1, 4.6 Hz, 2H), 7.20 (dd, J = 14.0, 7.0 Hz, 3H), 4.21 (dd, J = 7.5, 3.9 Hz, 1H), 2.93-2.80 (m, 3H), 2.73 (dt, J = 13.6, 8.2 Hz, 2H), 1.95-1.84 (m, 1H), 1.77 (tdd, J = 11.2, 6.2, 3.5 Hz, 1H); ^{13}C NMR (100 MHz, $CDCl_3$) δ 187.3, 141.6, 133.1, 131.0, 128.6, 128.5, 128.5, 125.9, 119.6, 91.9, 87.8, 66.9, 52.3, 38.0, 31.7; HRMS (EI): m/z $[M]^+$ calcd for $C_{19}H_{18}O_2$ 278.1307, observed 278.1306; HPLC: Chiralcel OJ-H column, IPA/hexane = 30/70, 1.0 mL/min, 210 nm; t_1 = 8.8 [(S)-**11**]; 99.9% *ee*.

HPLC chromatogram of (S)-**11**

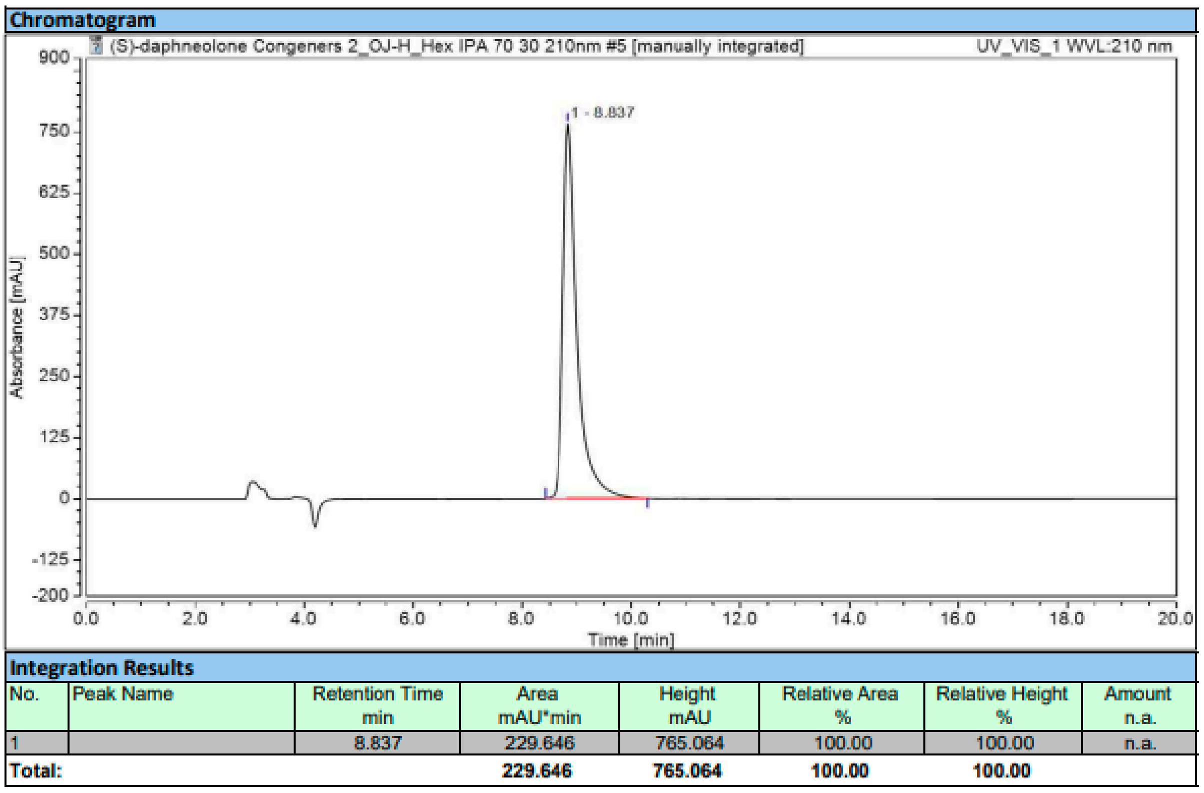

Integration Results

No.	Peak Name	Retention Time min	Area mAU*min	Height mAU	Relative Area %	Relative Height %	Amount n.a.
1		8.837	229.646	765.064	100.00	100.00	n.a.
**Total:**			**229.646**	**765.064**	**100.00**	**100.00**	

References

1. (a) Le Sann, C.; Muñoz, D. M.; Saunders, N.; Simpson, T. J.; Smith, D. I.; Soulas, F.; Watts, P.; Willis, C. L. *Org. Biomol. Chem.* **2005**, *3*, 1719– 1728. (b) Le Sann C.; Simpson T. J.; Smith D. I.; Watts P.; Willis C. L. *Tetrahedron Lett.* **1999**, *40*, 4093-4096.

2. Shibahara, F.; Fukunaga, T.; Murai, T. *Org. Lett.* **2018**, 20, *18*, 5826.

3. Tian, B.; Li, X.; Chen, P.; Liu, G. *Angew. Chem., Int. Ed.* **2021**, *60*, 14881.

4. (a) Fang, Z.; Wills, M. *J. Org. Chem.* **2013**, *78*, 8594. (b) Baker-Glenn, C.; Hodnett, N.; Reiter, M.; Ropp, S.; Ancliff, R.; Gouverneur, V. *J. Am. Chem. Soc.* **2005**, *127*, 1481.

5. Denniff, P.; Macleod, I.; Whiting, D. A. *J. Chem. Soc., Perkin Trans. 1*, **1981**, 82.

6. (a) Kogiso, S.; Hosozawa, S.; Wada, K.; Munakata, K *Phytochemistry* **1974**, *13*, 2332. (b) Wang, L.-B.; Dong, N.-W.; Wu, Z.-H.; Wu, L.-J. *J. Asian Nat. Prod. Res*. **2012**, *14*, 1020.

7. Xu, X.; Peng, L.; Chang, X.; Guo, C. *J. Am. Chem. Soc.* **2021**, *143*, 21048.

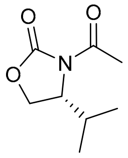

(*R*)-**1**

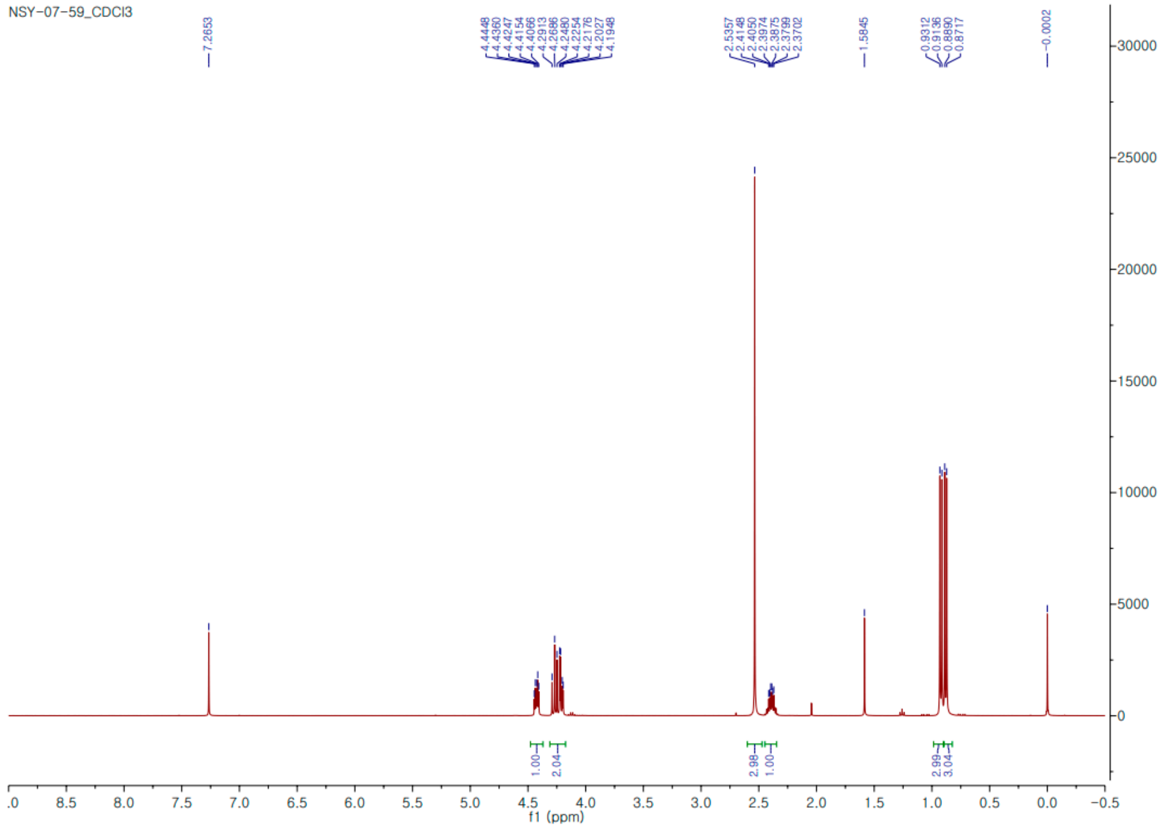

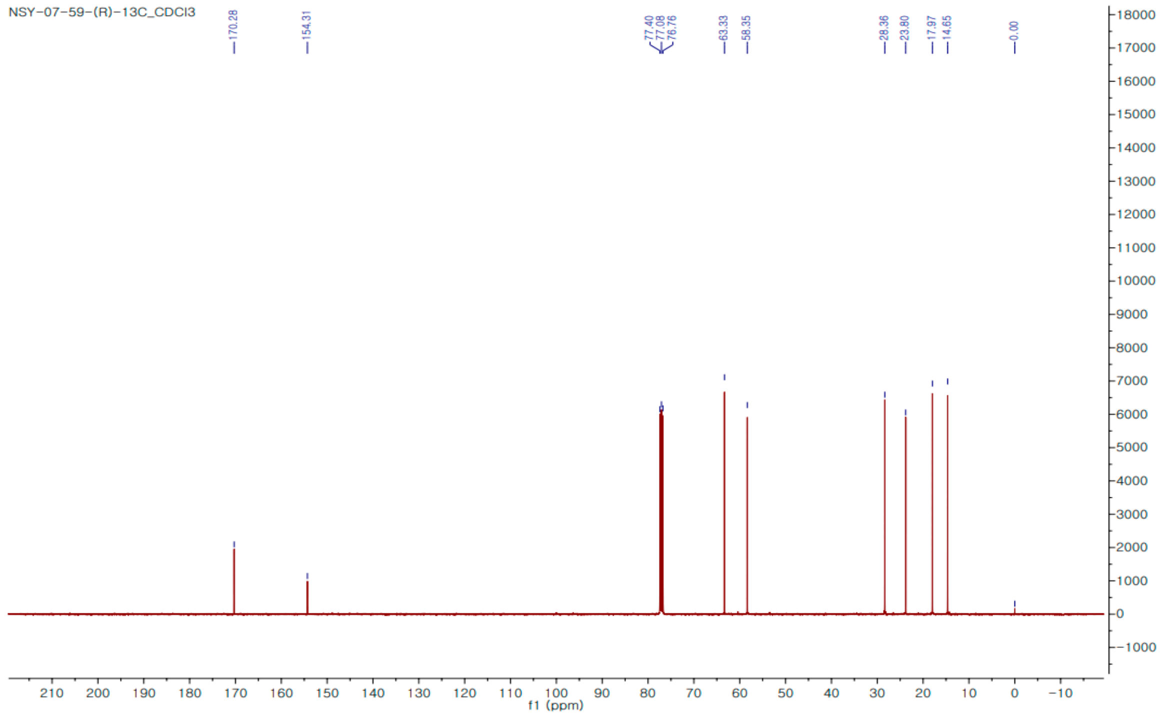

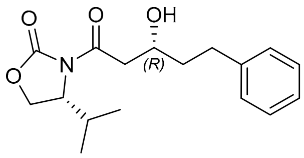
O
O
OH
N
O
(R)

(3'*R*,4*R*)-**2**

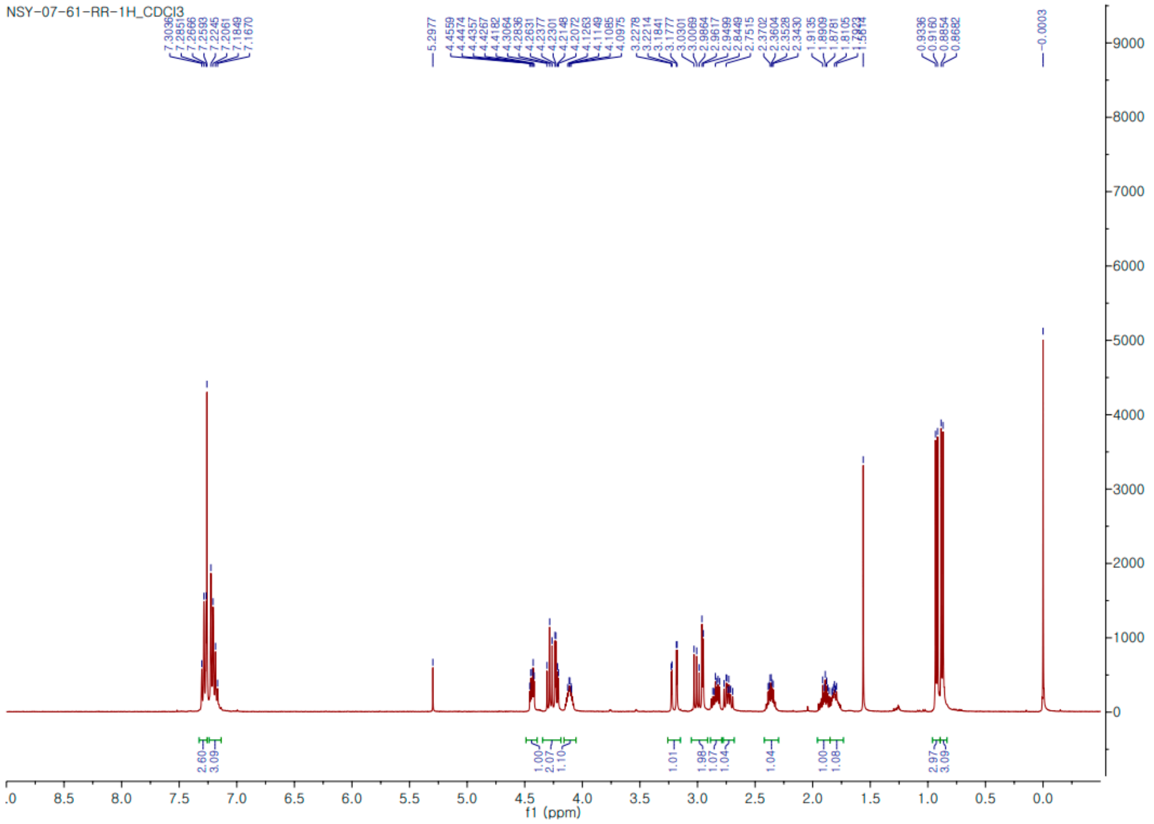
NSY-07-61-RR-1H_CDCl3
7.3036
7.2851
7.2666
7.2593
7.2245
7.2061
7.1849
7.1670
5.2977
4.4559
4.4474
4.4357
4.4267
4.4182
4.3064
4.2836
4.2631
4.2377
4.2301
4.2148
4.2072
4.1263
4.1149
4.1085
4.0975
3.2278
3.2214
3.1841
3.1777
3.0301
3.0069
2.9864
2.9617
2.9499
2.8449
2.7515
2.3702
2.3604
2.3528
2.3430
1.9135
1.8909
1.8781
1.8105
0.9336
0.9160
0.8854
0.8682
-0.0003
2.60
3.09
1.00
2.07
1.10
1.01
1.98
1.07
1.04
1.04
1.00
1.08
2.97
3.09
f1 (ppm)

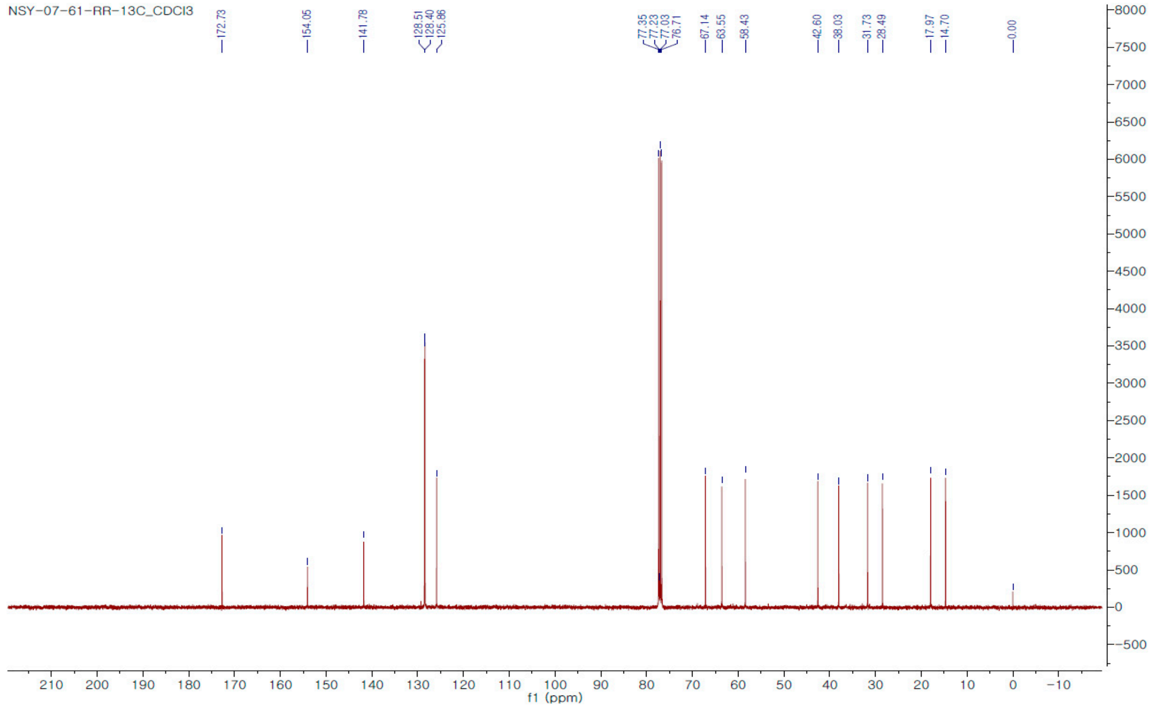
NSY-07-61-RR-13C_CDCl3
172.73
154.05
141.78
128.51
128.40
125.86
77.35
77.23
77.03
76.71
67.14
63.55
58.43
42.60
38.03
31.73
28.49
17.97
14.70
0.00
f1 (ppm)

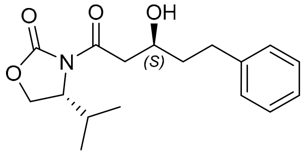

(3’*S*,4*R*)-**2**

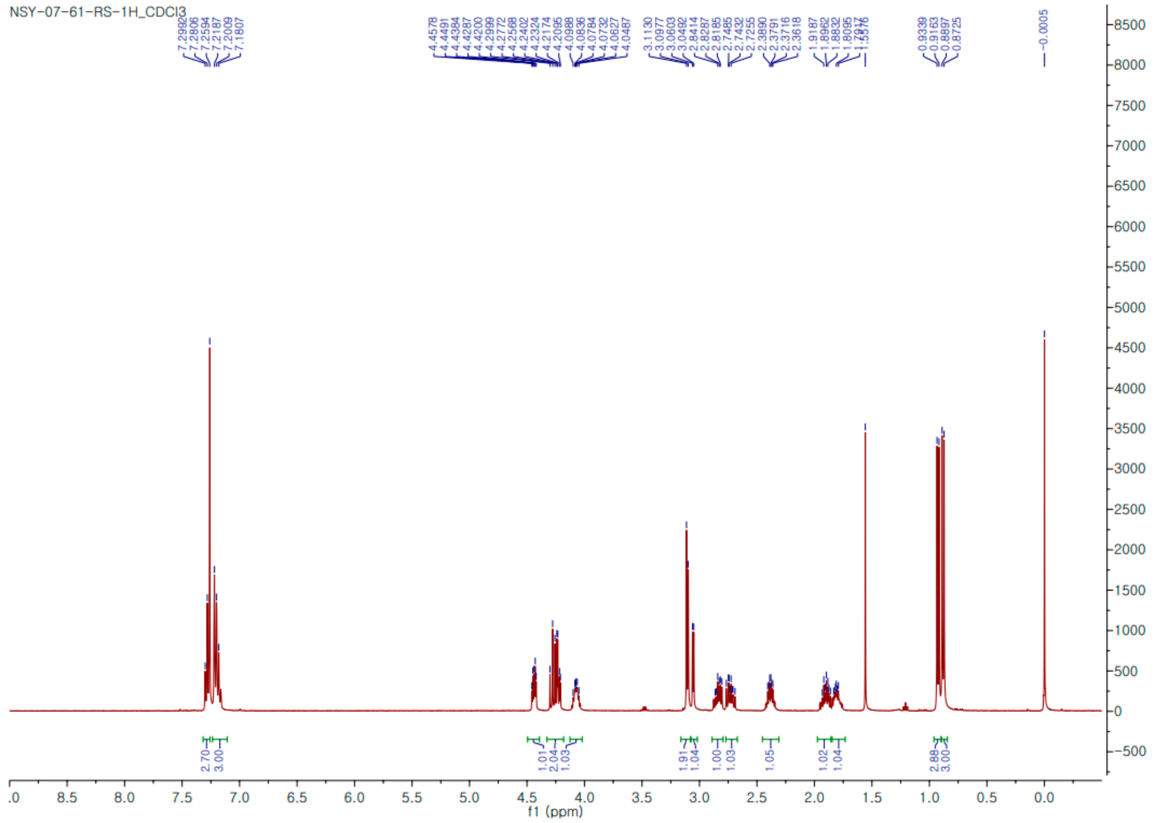
NSY-07-61-RS-1H_CDCl3
f1 (ppm)

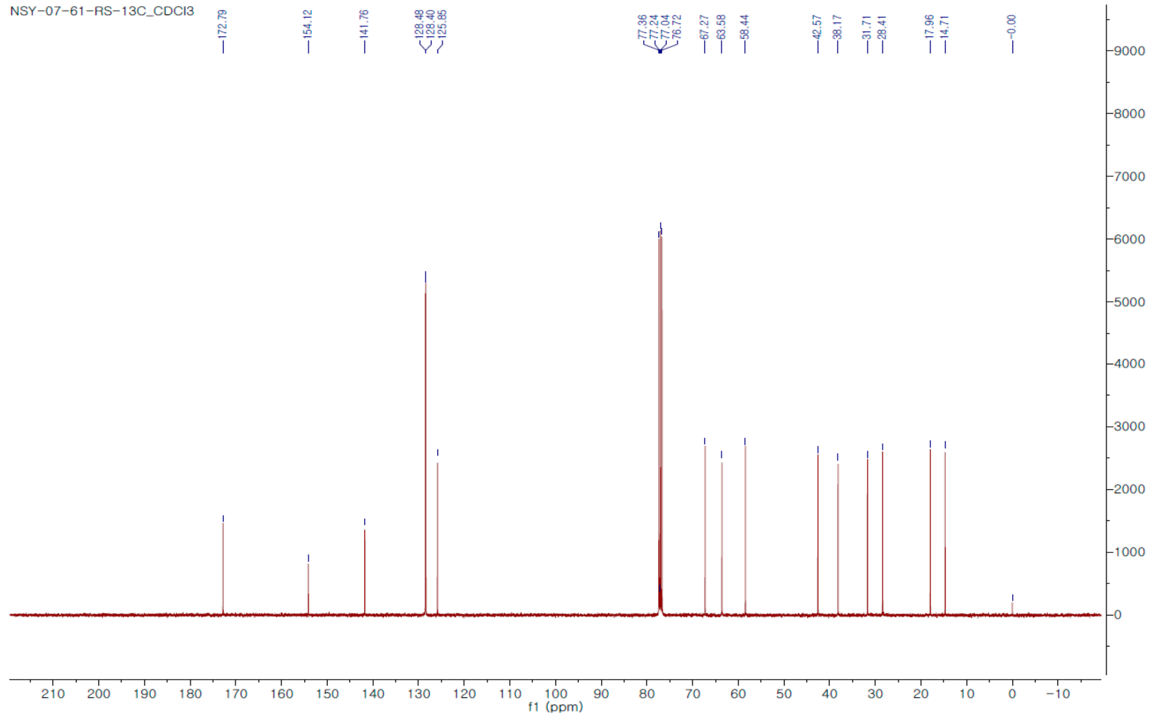
NSY-07-61-RS-13C_CDCl3
172.79
154.12
141.76
128.48
128.40
125.85
77.36
77.24
77.04
76.72
67.27
63.58
58.44
42.57
38.17
31.71
28.41
17.96
14.71
-0.00
f1 (ppm)

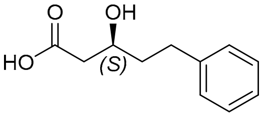

(*S*)-**3**

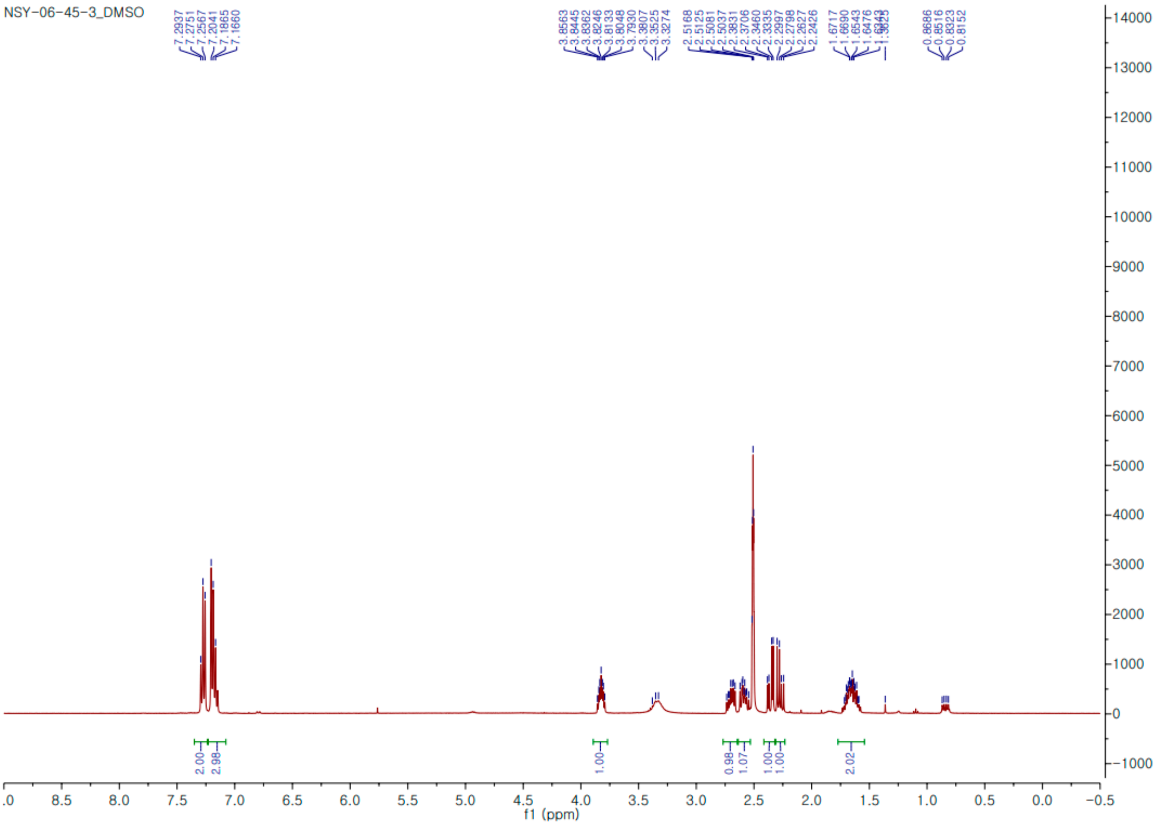

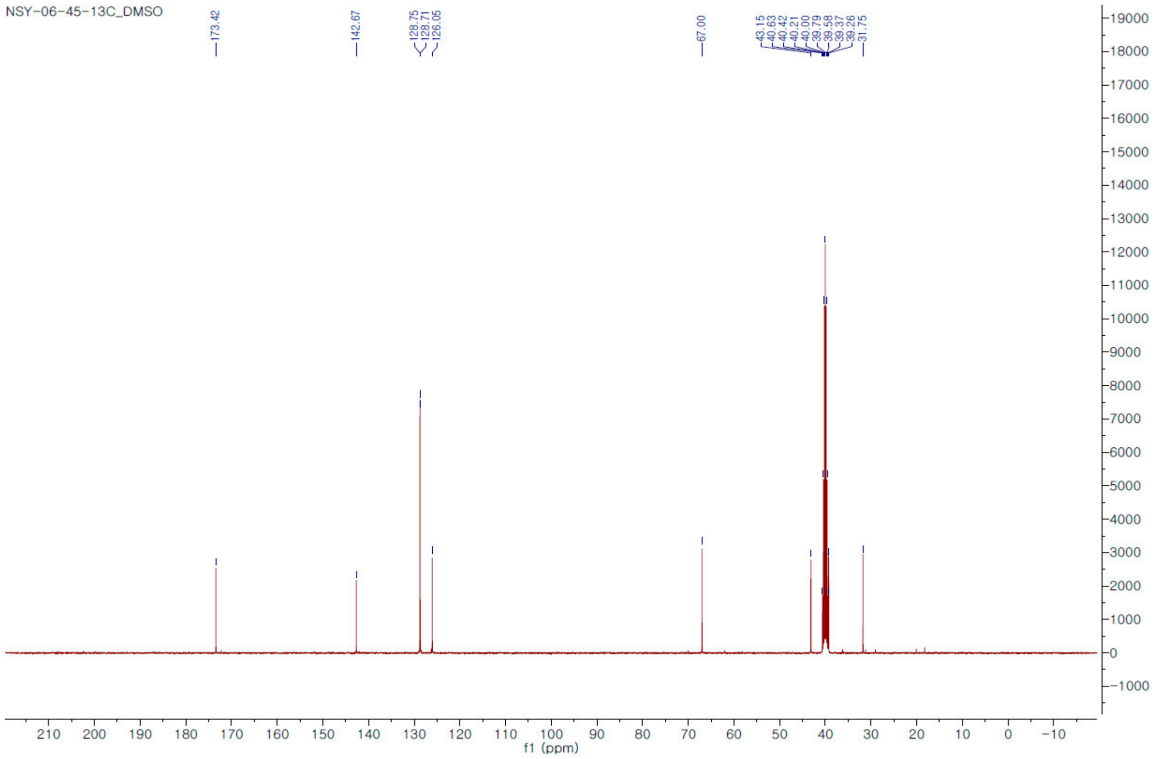

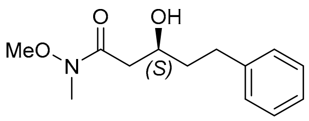
MeO
N
O
OH
(S)

(*S*)-**4**

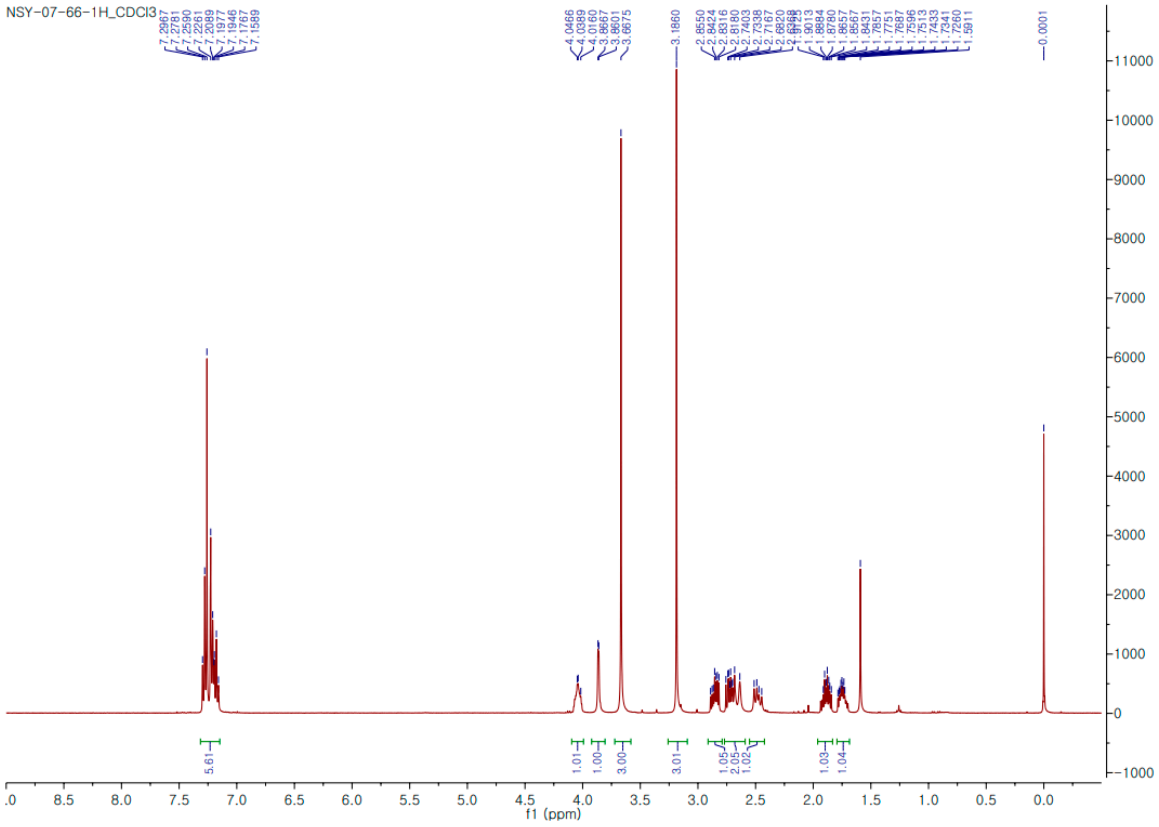
NSY-07-66-1H_CDCl3
f1 (ppm)

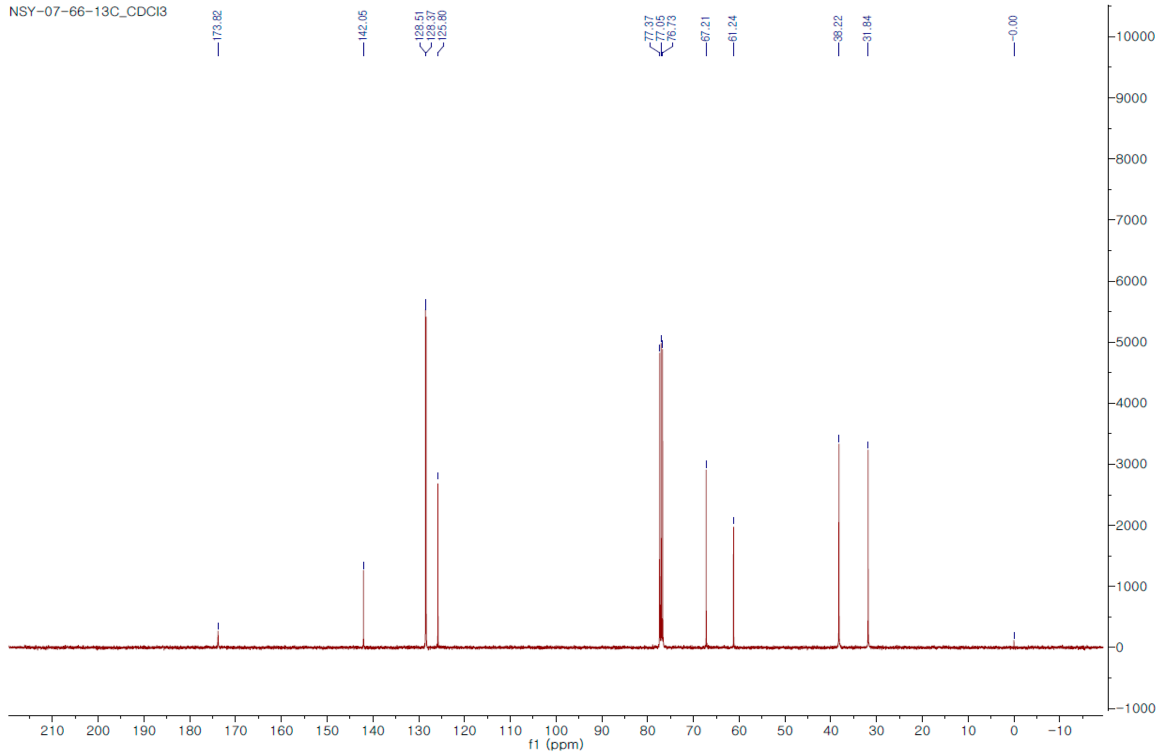
NSY-07-66-13C_CDCl3
173.82
142.05
128.51
128.37
125.80
77.37
77.05
76.73
67.21
61.24
38.22
31.84
-0.00
f1 (ppm)

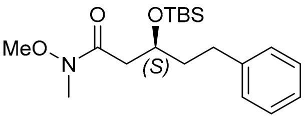

(*S*)-**5**

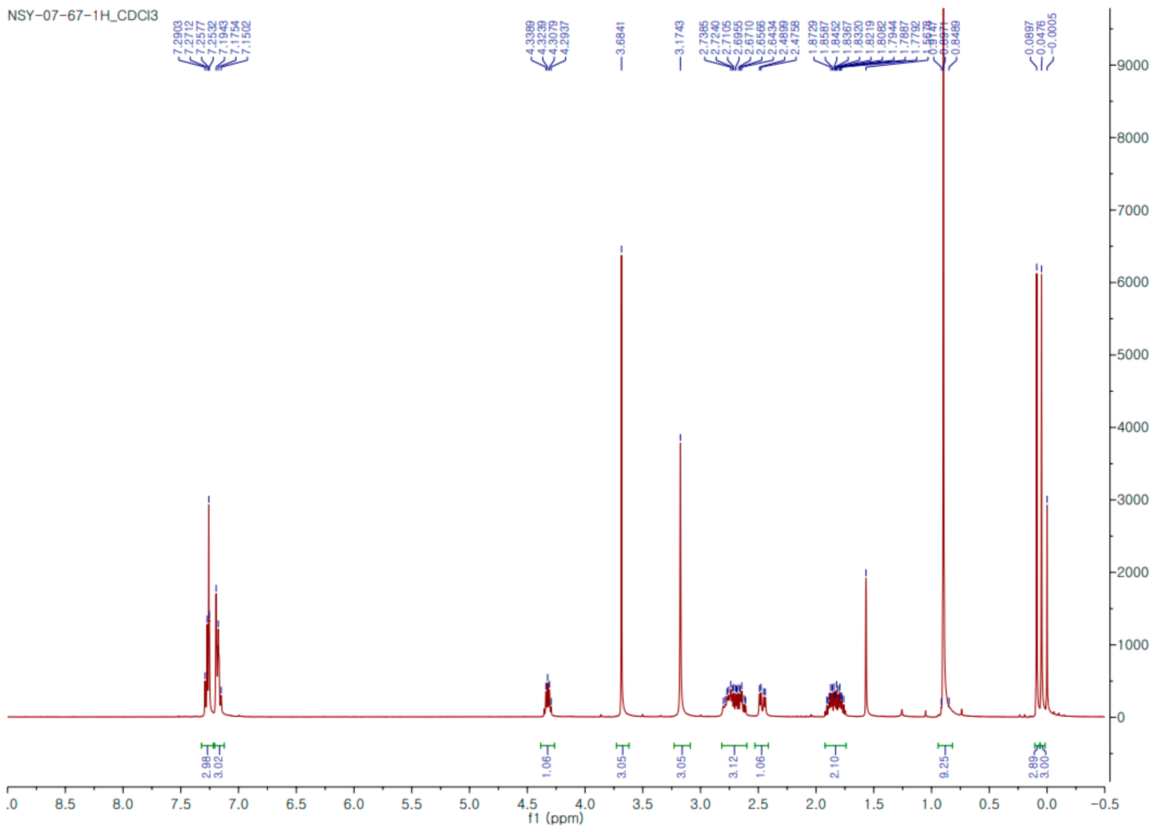

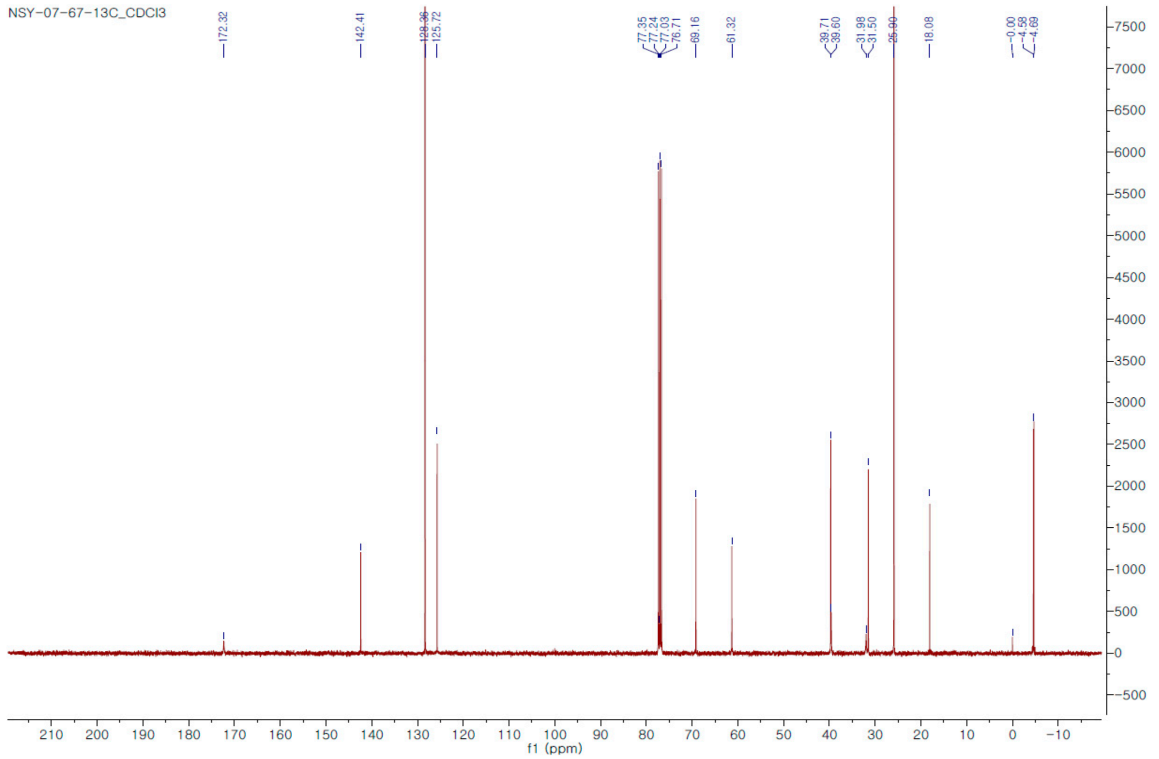

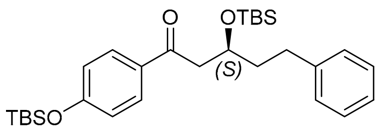

(*S*)-**6**

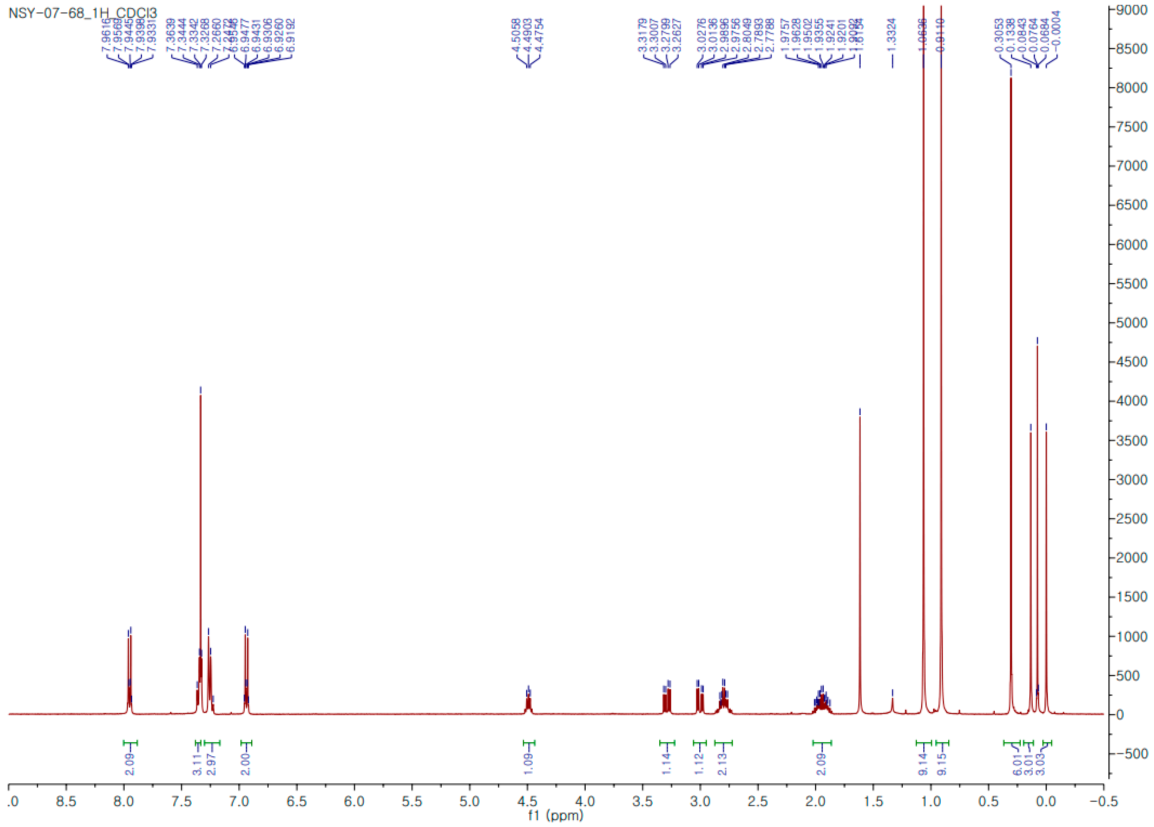

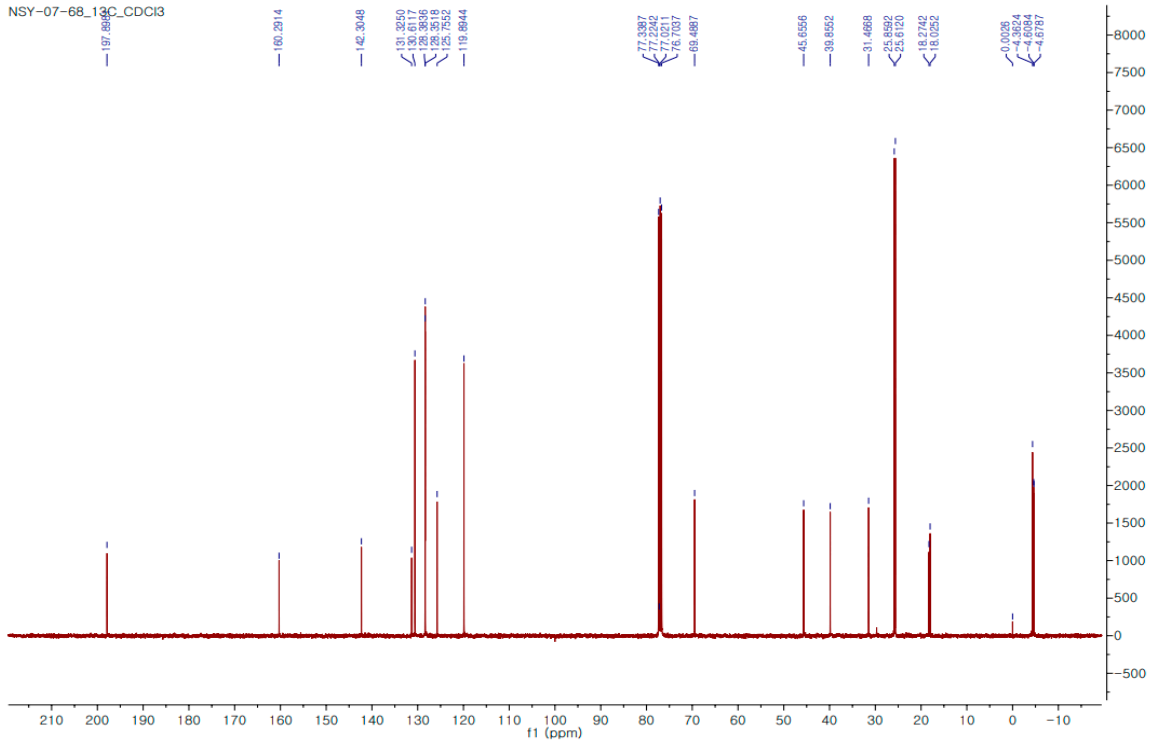

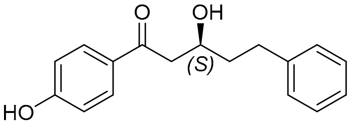
O
OH
(S)
HO

(*S*)-**7**

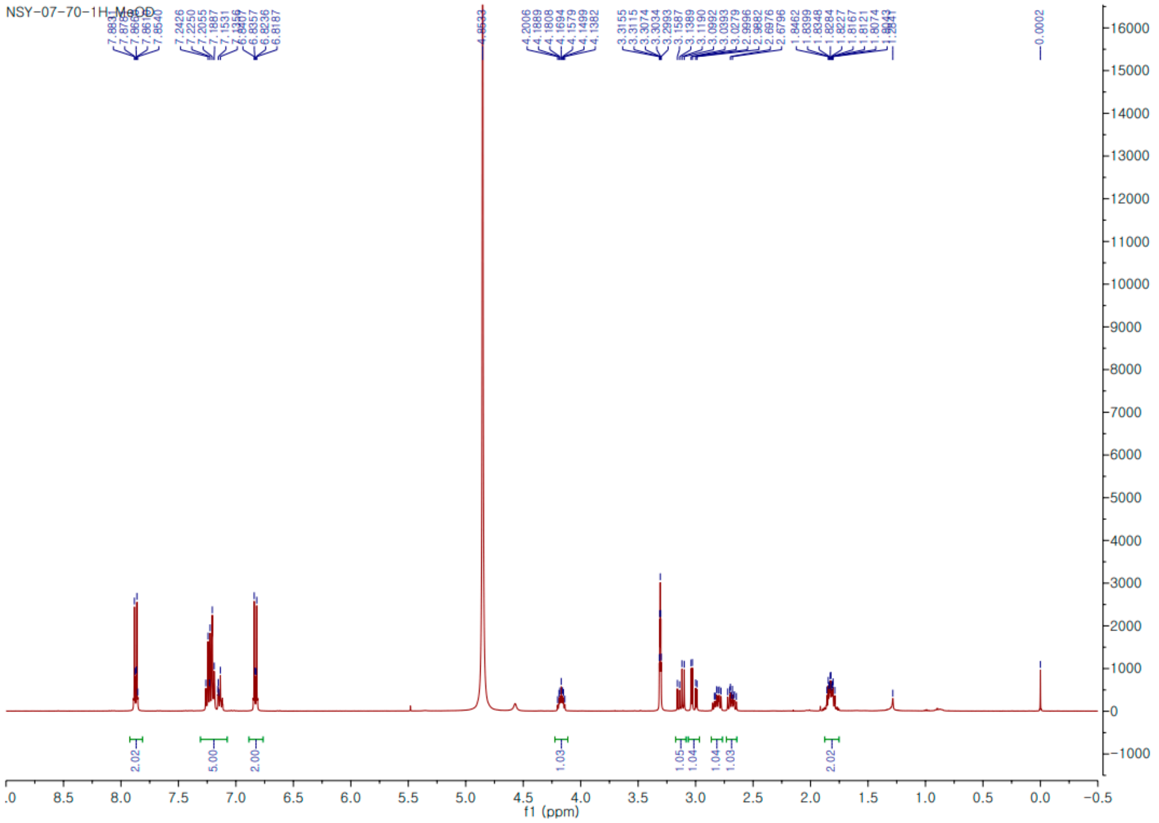
NSY-07-70-1H_MeOD
f1 (ppm)

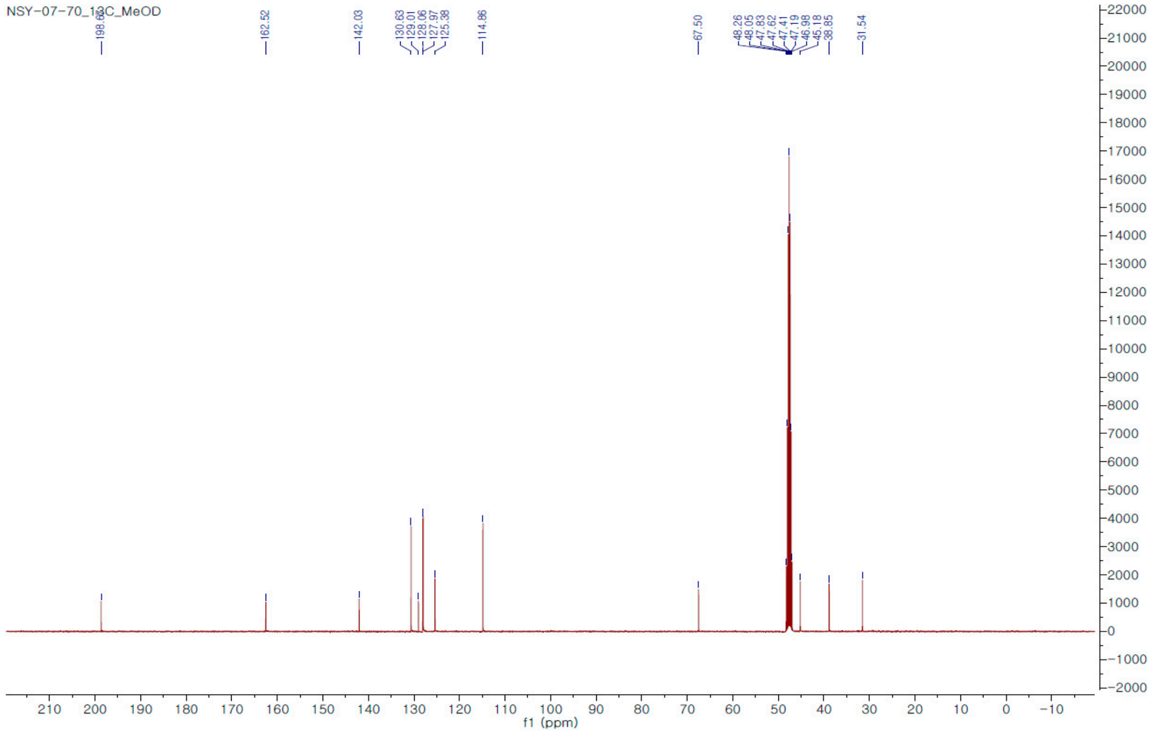
NSY-07-70_13C_MeOD
162.52
142.03
130.63
129.01
128.06
127.97
125.38
114.86
67.50
48.26
48.05
47.83
47.62
47.41
47.19
46.98
45.18
38.85
31.54
f1 (ppm)

HRMS of (*S*)-**7**

KRICT 한국화학연구원

별첨1. 시료 MM1의 분석결과.

Data : 1-20-001 Date : 20-Jan-2025 10:13
Instrument : MStation
Sample : MM1
Note :
Inlet : Direct Ion Mode : EI+
RT : 1.54 min Scan# : 47
Elements : C 17/0, H 18/0, O 3/0
Mass Tolerance : 1000ppm, 5mmu if m/z < 5, 10mmu if m/z > 10
Unsaturation (U.S.) : -0.5 - 20.0

	Observed m/z	Int%	Err[ppm / mmu]	U.S.	Composition
1	270.1250	43.65	-2.2 / -0.6	9.0	C17 H18 O3

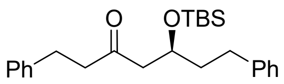
O
OTBS
Ph
Ph

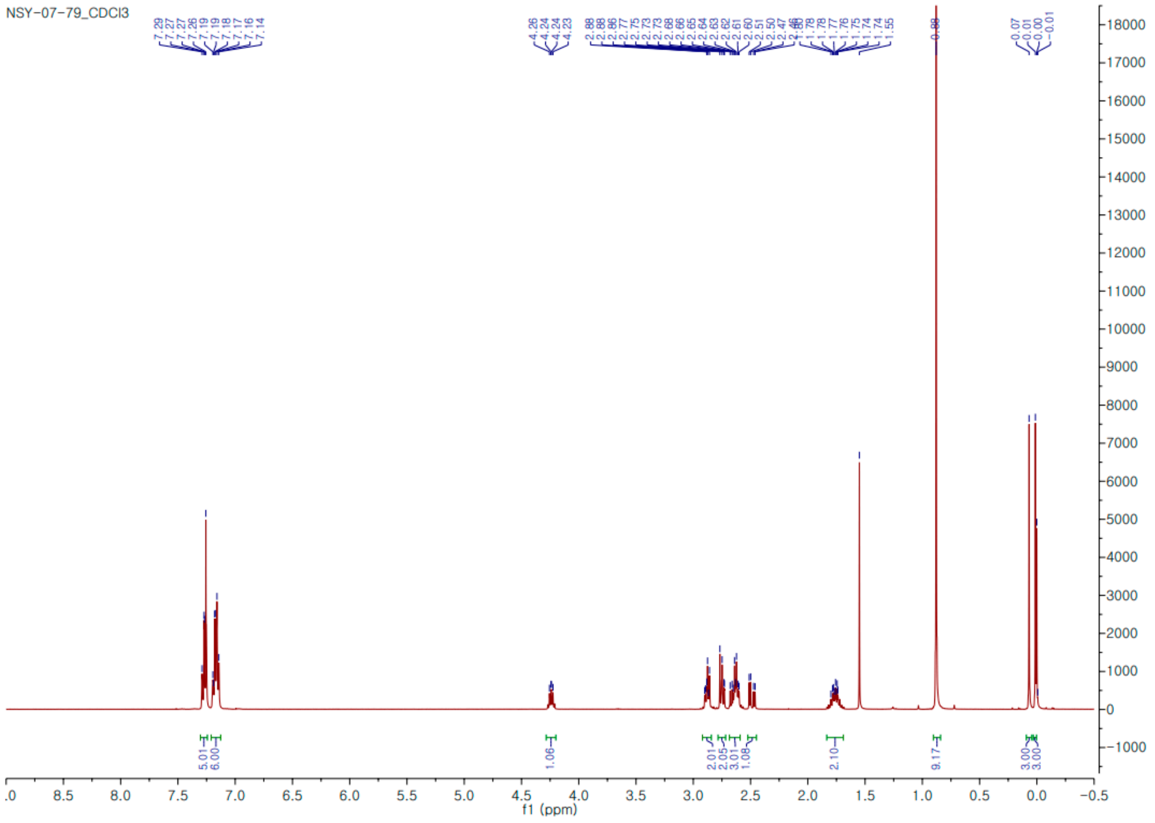
NSY-07-79_CDCl3
f1 (ppm)

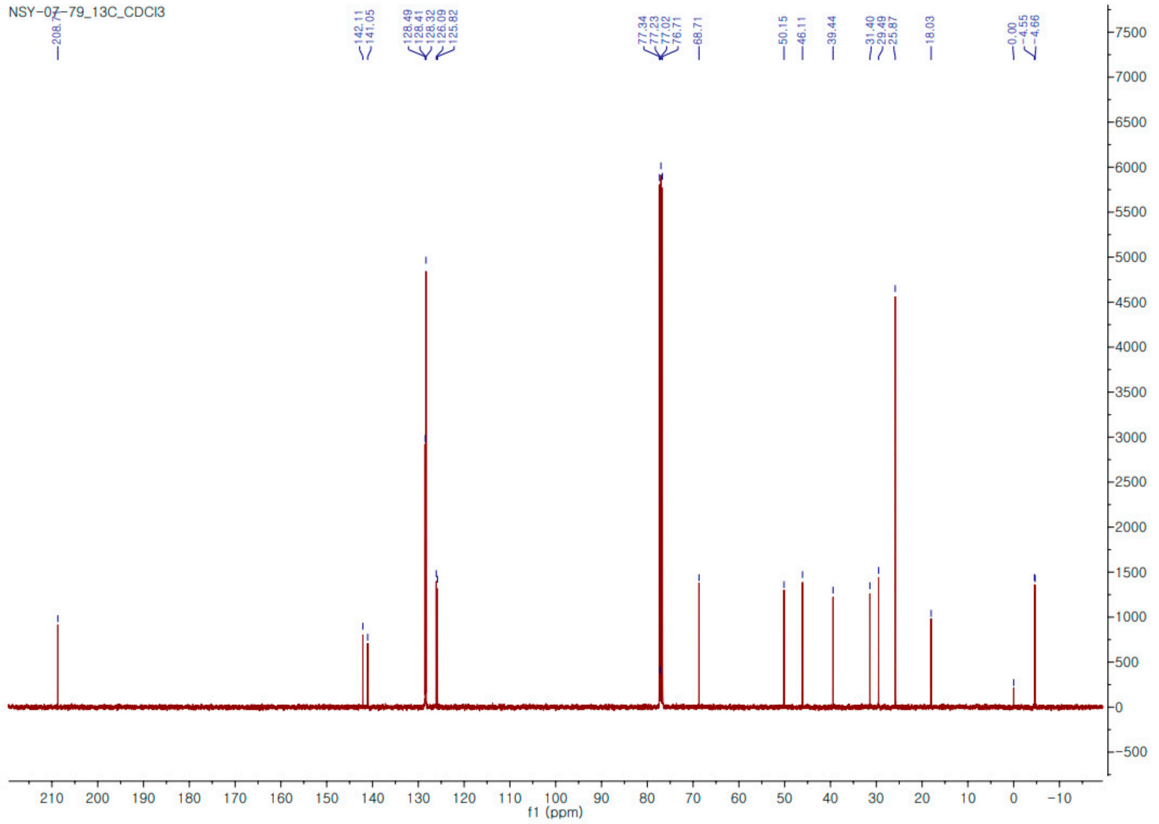
NSY-07-79_13C_CDCl3
f1 (ppm)

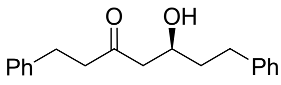

(*S*)-**9**

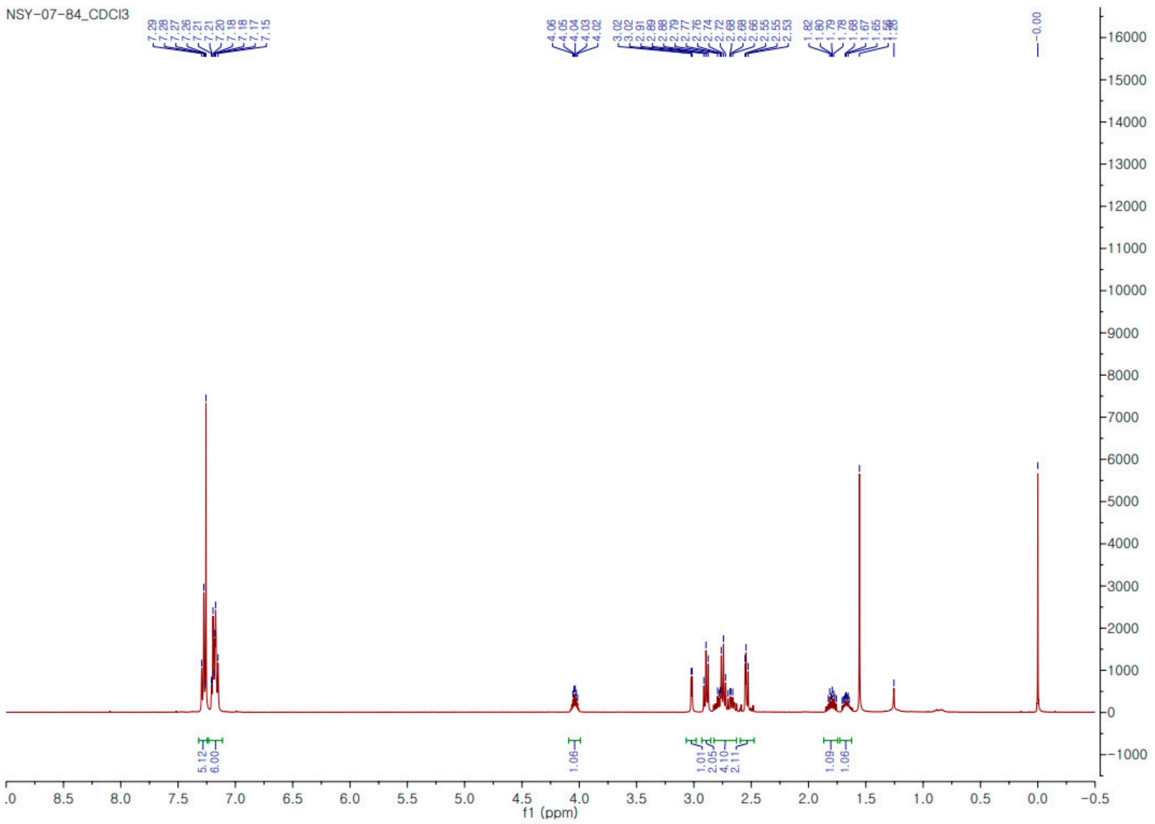

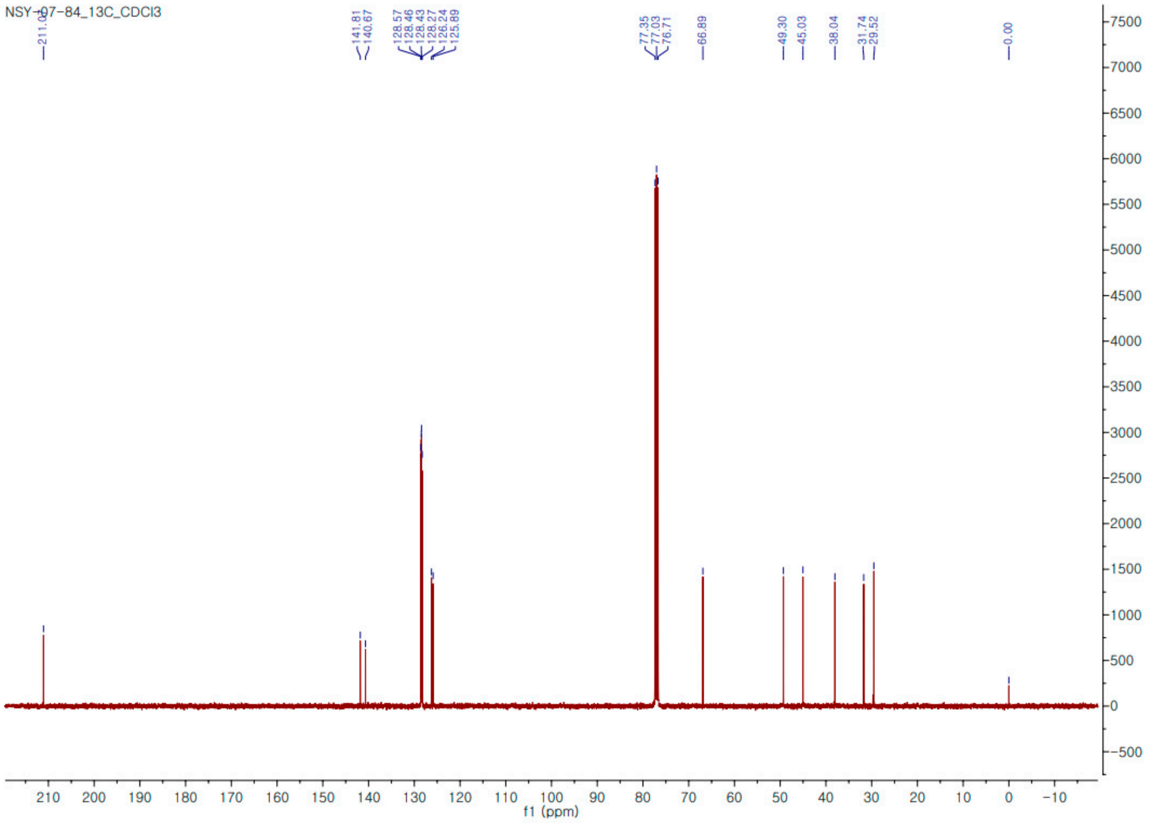

HRMS of (*S*)-**9**

KRICT 한국화학연구원

별첨2. 시료 MM2의 분석결과.

Data : 1-20-002 Date : 20-Jan-2025 10:23
Instrument : MStation
Sample : MM2
Note :
Inlet : Direct Ion Mode : EI+
RT : 0.37 min Scan# : 12
Elements : C 19/0, H 22/0, O 2/0
Mass Tolerance : 1000ppm, 5mmu if m/z < 5, 10mmu if m/z > 10
Unsaturation (U.S.) : -0.5 - 20.0

	Observed m/z	Int%	Err[ppm / mmu]	U.S.	Composition
1	282.1615	3.13	-1.7 / -0.5	9.0	C19 H22 O2

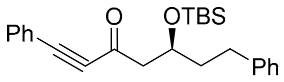
Ph
O
OTBS
Ph

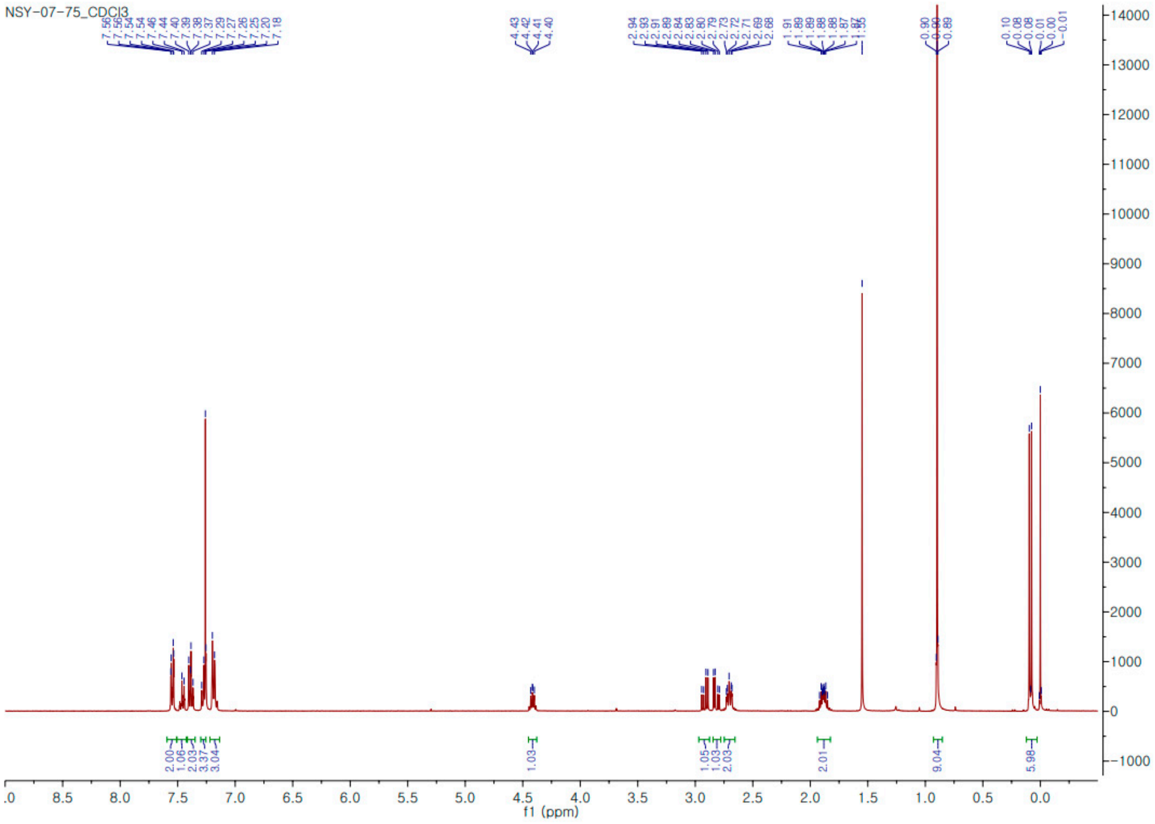
NSY-07-75_CDCl3
7.56 7.55 7.54 7.54 7.46 7.44 7.40 7.39 7.38 7.37 7.29 7.27 7.26 7.25 7.20 7.18
4.43 4.42 4.41 4.40
2.94 2.93 2.91 2.89 2.84 2.83 2.80 2.79 2.73 2.72 2.71 2.69 2.68
1.91 1.89 1.89 1.88 1.88 1.87 1.57 1.55
0.90 0.90 0.89
0.10 0.08 0.08 0.01 0.00 -0.01
2.00 1.06 2.03 2.37 3.04
1.03
1.05 1.03 2.03
2.01
9.04
5.98
f1 (ppm)

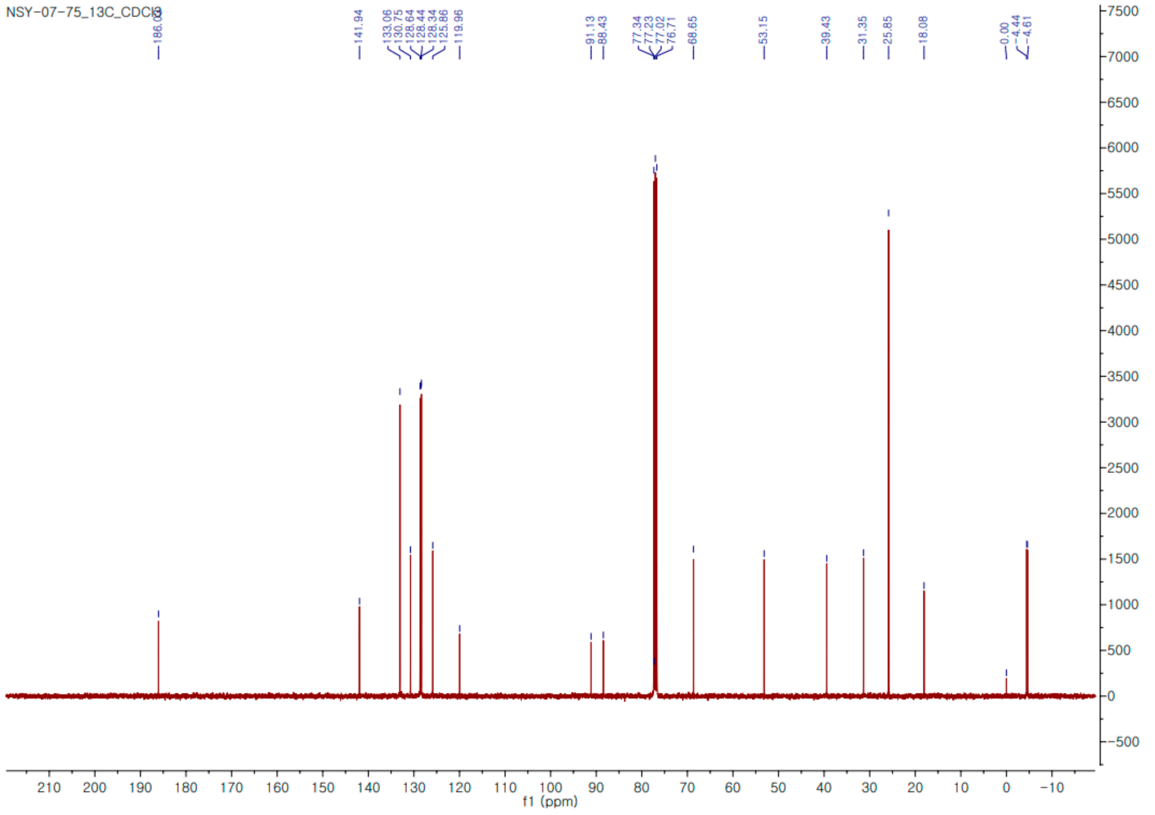
NSY-07-75_13C_CDCl3
186.08
141.94
133.06 130.75 128.64 128.44 128.34 125.86 119.96
91.13 88.43
77.34 77.23 77.02 76.71
68.65
53.15
39.43
31.35
25.85
18.08
0.00 -4.44 -4.61
f1 (ppm)

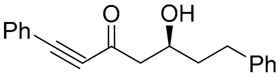

(*S*)-**11**

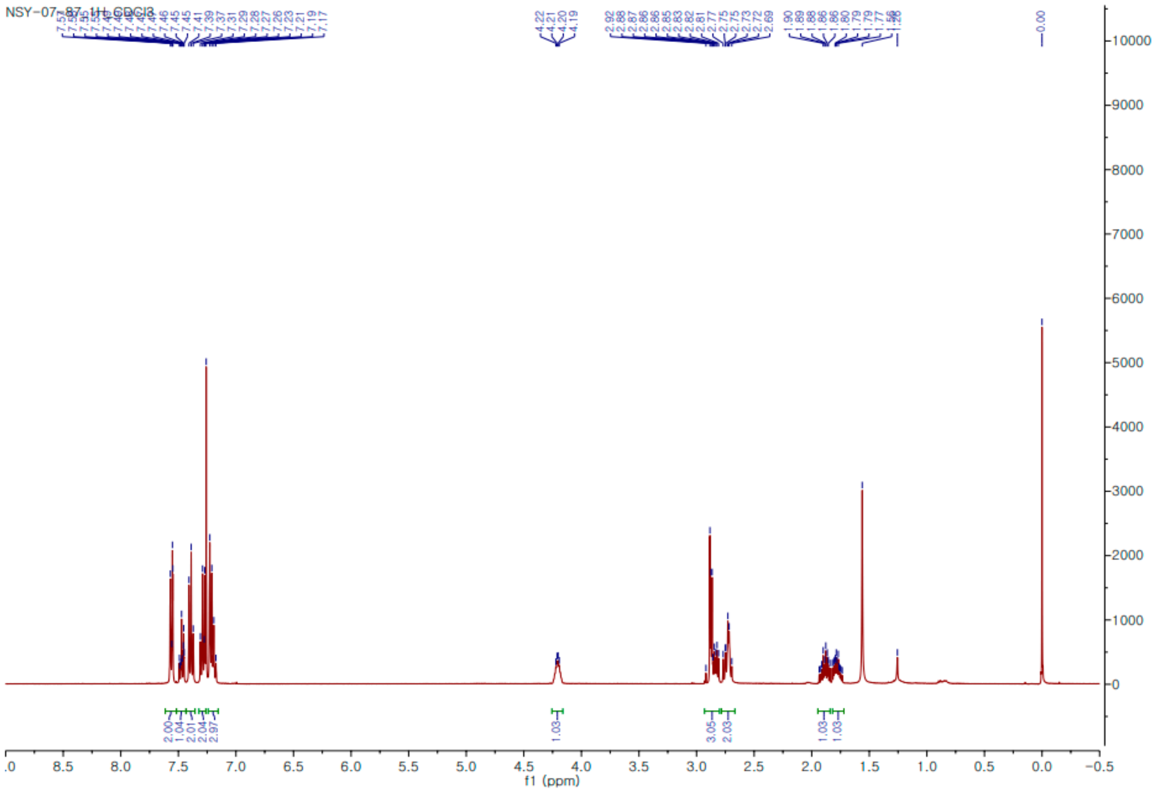

NSY-07-87_1H_CDCl3
f1 (ppm)

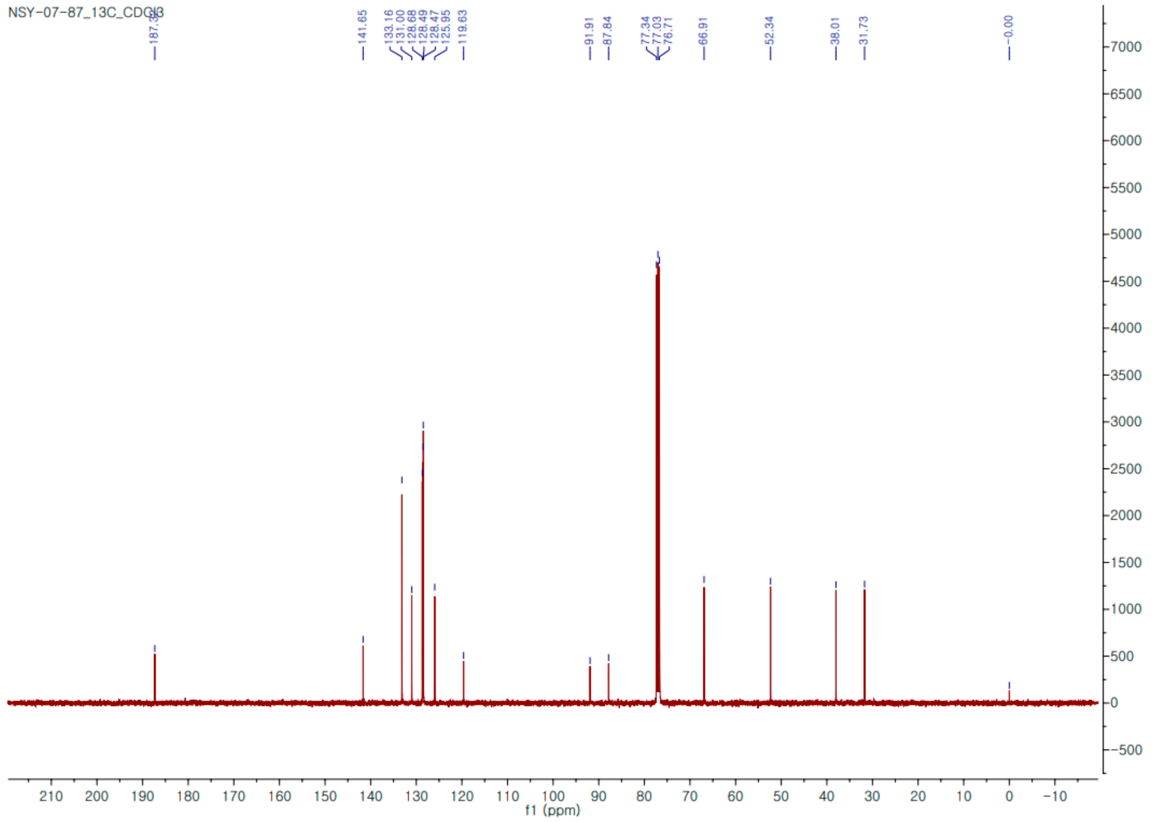

NSY-07-87_13C_CDCl3
141.65
133.16
131.00
128.68
128.49
128.47
125.95
119.63
91.91
87.84
77.34
77.03
76.71
66.91
52.34
38.01
31.73
-0.00
f1 (ppm)

HRMS of (*S*)-**11**

KRICT 한국화학연구원

별첨3. 시료 MM3의 분석결과.

Data : 1-20-003   Date : 20-Jan-2025 10:30
Instrument : MStation
Sample : MM3
Note :
Inlet : Direct   Ion Mode : EI+
RT : 1.10 min   Scan# : 34
Elements : C 19/0, H 18/0, O 2/0
Mass Tolerance : 1000ppm, 5mmu if m/z < 5, 10mmu if m/z > 10
Unsaturation (U.S.) : -0.5 - 20.0

	Observed m/z	Int%	Err[ppm / mmu]	U.S.	Composition
1	278.1306	100.00	-0.3 / -0.1	11.0	C19 H18 O2
